# Histopathology-based protein multiplex generation using deep learning

**DOI:** 10.1038/s42256-025-01074-y

**Published:** 2025-08-04

**Authors:** Sonali Andani, Boqi Chen, Joanna Ficek-Pascual, Simon Heinke, Ruben Casanova, Bernard Friedrich Hild, Bettina Sobottka, Bernd Bodenmiller, Tumor Profiler Consortium, Rudolf Aebersold, Melike Ak, Faisal S. Al-Quaddoomi, Silvana I. Albert, Jonas Albinus, Ilaria Alborelli, Sonali Andani, Per-Olof Attinger, Marina Bacac, Daniel Baumhoer, Beatrice Beck-Schimmer, Niko Beerenwinkel, Christian Beisel, Lara Bernasconi, Anne Bertolini, Bernd Bodenmiller, Ximena Bonilla, Lars Bosshard, Byron Calgua, Ruben Casanova, Stéphane Chevrier, Natalia Chicherova, Ricardo Coelho, Maya D’Costa, Esther Danenberg, Natalie R. Davidson, Monica-Andreea Baciu-Drăgan, Reinhard Dummer, Stefanie Engler, Martin Erkens, Katja Eschbach, Cinzia Esposito, André Fedier, Pedro F. Ferreira, Joanna Ficek-Pascual, Anja L. Frei, Bruno Frey, Sandra Goetze, Linda Grob, Gabriele Gut, Detlef Günther, Pirmin Haeuptle, Viola Heinzelmann-Schwarz, Sylvia Herter, Rene Holtackers, Tamara Huesser, Alexander Immer, Anja Irmisch, Francis Jacob, Andrea Jacobs, Tim M. Jaeger, Katharina Jahn, Alva R. James, Philip M. Jermann, André Kahles, Abdullah Kahraman, Viktor H. Koelzer, Werner Kuebler, Jack Kuipers, Christian P. Kunze, Christian Kurzeder, Kjong-Van Lehmann, Mitchell Levesque, Ulrike Lischetti, Flavio C. Lombardo, Sebastian Lugert, Gerd Maass, Markus G. Manz, Philipp Markolin, Martin Mehnert, Julien Mena, Julian M. Metzler, Nicola Miglino, Emanuela S. Milani, Holger Moch, Simone Muenst, Riccardo Murri, Charlotte KY Ng, Stefan Nicolet, Marta Nowak, Monica Nunez Lopez, Patrick GA Pedrioli, Lucas Pelkmans, Salvatore Piscuoglio, Michael Prummer, Laurie Prélot, Natalie Rimmer, Mathilde Ritter, Christian Rommel, Mara L. Rosano-González, Gunnar Rätsch, Natascha Santacroce, Jacobo Sarabia del Castillo, Ramona Schlenker, Petra C. Schwalie, Severin Schwan, Tobias Schär, Gabriela Senti, Wenguang Shao, Franziska Singer, Sujana Sivapatham, Berend Snijder, Bettina Sobottka, Vipin T. Sreedharan, Stefan Stark, Daniel J. Stekhoven, Tanmay Tanna, Alexandre PA Theocharides, Tinu M. Thomas, Markus Tolnay, Vinko Tosevski, Nora C. Toussaint, Mustafa A. Tuncel, Marina Tusup, Audrey Van Drogen, Marcus Vetter, Tatjana Vlajnic, Sandra Weber, Walter P. Weber, Rebekka Wegmann, Michael Weller, Fabian Wendt, Norbert Wey, Andreas Wicki, Mattheus HE Wildschut, Bernd Wollscheid, Shuqing Yu, Johanna Ziegler, Marc Zimmermann, Martin Zoche, Gregor Zuend, Viktor H. Koelzer, Gunnar Rätsch

**Affiliations:** 1 Department of Computer Science, ETH Zurich, Zurich, Switzerland; 2 Computational and Translational Pathology Group, Department of Biomedical Engineering, University of Basel, Basel, Switzerland; 3 Swiss Institute of Bioinformatics, Zurich, Switzerland; 4 AI Center, ETH Zurich, Zurich, Switzerland; 5 Computer Vision Laboratory, Department of Information Technology and Electrical Engineering, ETH Zurich, Zurich, Switzerland; 6 Department of Quantitative Biomedicine, University of Zurich, Zurich, Switzerland; 7 Department of Pathology and Molecular Pathology, University Hospital Zurich, University of Zurich, Zurich, Switzerland; 8 Institute of Medical Genetics and Pathology, University Hospital Basel, Basel, Switzerland; 9 Medical Informatics Unit, University Hospital Zurich, Zurich, Switzerland; 10 Institute of Molecular Systems Biology, Department of Biology, ETH Zurich, Zurich, Switzerland; 11 Department of Dermatology, University Hospital Zurich, Zurich, Switzerland; 12 NEXUS Personalized Health Technologies, ETH Zurich, Zurich, Switzerland; 13 SIB Swiss Institute of Bioinformatics, Lausanne, Switzerland; 14 Department of Health Sciences and Technology, ETH Zurich, Zurich, Switzerland; 15 Department of Computer Science, Institute of Machine Learning, ETH Zurich, Zurich, Switzerland; 16 Biomedical Informatics, University Hospital Zurich, Zurich, Switzerland; 17 Department of Pathology and Molecular Pathology, University Hospital Zurich, Zurich, Switzerland; 18 F. Hoffmann-La Roche Ltd, Basel, Switzerland; 19 Roche Pharmaceutical Research and Early Development, Roche Innovation Center Zurich, Schlieren, Switzerland; 20 VP Medicine, University of Zurich, Zurich, Switzerland; 21 Department of Biosystems Science and Engineering, ETH Zurich, Basel, Switzerland; 22 Clinical Trials Center, University Hospital Zurich, Zurich, Switzerland; 23 Institute of Molecular Health Sciences, ETH Zurich, Zurich, Switzerland; 24 Department of Biomedicine, University Hospital Basel and University of Basel, Basel, Switzerland; 25 Institute of Molecular Life Sciences, University of Zurich, Zurich, Switzerland; 26 Roche Pharmaceutical Research and Early Development, Roche Innovation Center Basel, Basel, Switzerland; 27 Biomedicine PhD Program, Life Science Zurich Graduate School, Zurich, Switzerland; 28 Roche Diagnostics GmbH, Penzberg, Germany; 29 Department of Chemistry and Applied Biosciences, ETH Zurich, Zurich, Switzerland; 30 Medical University Clinic, Cantonal Hospital Baselland, Liestal, Switzerland; 31 Gynecological Cancer Center, University Hospital Basel, Basel, Switzerland; 32 Max Planck ETH Center for Learning Systems, Stuttgart, Germany; 33 Faculty of Medicine, University of Zurich, Zurich, Switzerland; 34 University Hospital Basel, Basel, Switzerland; 35 Department of Information and Communication Technology, University Hospital Basel, Basel, Switzerland; 36 Brustzentrum, University Hospital Basel, Basel, Switzerland; 37 Cancer Research Center Cologne-Essen, University Hospital Cologne, Cologne, Germany; 38 Center for Integrated Oncology Aachen (CIO-A), Aachen, Germany; 39 Joint Research Center Computational Biomedicine, University Hospital RWTH Aachen, Aachen, Germany; 40 Department of Medical Oncology and Hematology, University Hospital Zurich, Zurich, Switzerland; 41 Department of Gynecology, University Hospital Zurich, Zurich, Switzerland; 42 Services and Support for Science IT, University of Zurich, Zurich, Switzerland; 43 Department of BioMedical Research, University of Bern, Bern, Switzerland; 44 Department of Biology, ETH Zurich, Zurich, Switzerland; 45 Roche Pharmaceutical Research and Early Development, Roche Innovation Center Munich, Roche Diagnostics GmbH, Penzberg, Germany; 46 Swiss Data Science Center, ETH Zurich, Zurich, Switzerland; 47 Brustzentrum & Tumorzentrum, University Hospital Basel, Basel, Switzerland; 48 Department of Surgery, Brustzentrum, University Hospital Basel and University of Basel, Basel, Switzerland; 49 Department of Neurology, University Hospital and University of Zurich, Zurich, Switzerland; 50 University Hospital Zurich, Zurich, Switzerland

**Keywords:** Computational models, Melanoma, Machine learning

## Abstract

Multiplexed protein imaging offers valuable insights into interactions between tumours and their surrounding tumour microenvironment, but its widespread use is limited by cost, time and tissue availability. Here we present HistoPlexer, a deep learning framework that generates spatially resolved protein multiplexes directly from standard haematoxylin and eosin (H&E) histopathology images. HistoPlexer jointly predicts multiple tumour and immune markers using a conditional generative adversarial architecture with custom loss functions designed to ensure pixel- and embedding-level similarity while mitigating slice-to-slice variations. A comprehensive evaluation of metastatic melanoma samples demonstrates that HistoPlexer-generated protein maps closely resemble real maps, as validated by expert assessment. They preserve crucial biological relationships by capturing spatial co-localization patterns among proteins. The spatial distribution of immune infiltration from HistoPlexer-generated protein multiplex enables stratification of tumours into immune subtypes. In an independent cohort, integration of HistoPlexer-derived features into predictive models enhances performance in survival prediction and immune subtype classification compared to models using H&E features alone. To assess broader applicability, we benchmarked HistoPlexer on publicly available pixel-aligned datasets from different cancer types. In all settings, HistoPlexer consistently outperformed baseline methods, demonstrating robustness across diverse tissue types and imaging conditions. By enabling whole-slide protein multiplex generation from routine H&E images, HistoPlexer offers a cost- and time-efficient approach to tumour microenvironment characterization with strong potential to advance precision oncology.

## Abstract (web summary)

HistoPlexer, a deep learning model, generates multiplexed protein expression maps from H&E images, capturing tumour–immune cell interactions. It outperforms baselines, enhances immune subtyping and survival prediction and offers a cost-effective tool for precision oncology.

## Main

Tumours are complex systems that acquire hallmark traits by establishing a supportive tumour microenvironment (TME) that facilitates tumorigenesis and metastasis^1,2^. Understanding cancer cell interactions with surrounding tissue provides critical insights into disease progression and therapeutic response^3–5^. Multiplexed immunohistochemistry and immunofluorescence (mIHC/IF) technologies, such as imaging mass cytometry (IMC), enable spatially resolved quantification of up to 40 protein markers, offering comprehensive visualization of tumour–TME interactions^4,6,7^. These technologies facilitate analysis of spatial cell distribution, phenotype co-localization and cellular interactions, which are valuable for clinical decision-making^4,5,8,9^. However, IMC is limited by low throughput, high cost and coverage restricted to small regions of interest (ROIs), hindering its broader clinical adoption.

In contrast, haematoxylin and eosin (H&E) staining remains the gold standard for cancer diagnosis in clinical practice due to its low cost, high throughput and entire tissue section coverage. H&E images reveal morphological features crucial for cancer grading, proliferation assessment and staging^10^. Recent deep learning advances have enabled the prediction of protein markers from H&E images, such as pan-cytokeratin for pancreatic cancer^11^, HER2 for breast cancer^12^ and Ki-67 for neuroendocrine and breast cancers^13^. One study predicted two markers, specifically one tumour and one immune marker^14^. Only a few studies have attempted to predict a set of protein markers, with a focus solely on either tumour^15,16^ or immune markers^17^, limiting their utility for investigation of tumour–TME interactions. These studies either use separate models per marker^15,17^ or lack quantitative validation of the advantages of multiplexed prediction with a single model^14,16,17^.

To address these limitations, we introduce HistoPlexer, a deep learning model that generates comprehensive protein multiplexes from H&E images. HistoPlexer simultaneously predicts 11 markers consisting of both tumour and immune markers, enabling an integrative visualization of tumour–host interactions. We train HistoPlexer on metastatic samples from the Tumor Profiler Study (TuPro)^18^ using paired H&E and IMC images from serial sections. Through rigorous quantitative evaluation, we demonstrate the importance of simultaneous marker prediction through improved model performance and enhanced spatial co-localization of markers. We validate the biological relevance of generated IMC images through cell typing and immune phenotyping, particularly in characterizing immune-hot (inflamed) and immune-cold (excluded/desert) tumours based on CD8^+^ T cell distributions. We demonstrate the generalizability of the trained HistoPlexer model on an independent patient cohort from the human skin cutaneous melanoma (SKCM) study of The Cancer Genome Atlas (TCGA) project^19^. Experiments on two public multiplexed datasets^14,20^ further validate its adaptability across various cancer types.

Our results demonstrate that HistoPlexer generates high-quality IMC images that closely align with real data distributions. These multiplexes enable precise immune phenotyping through spatial analysis of tumour–immune cell interactions, particularly in distinguishing immune-hot and -cold subtypes. Simultaneous marker prediction preserves biologically meaningful relationships. Furthermore, by augmenting H&E whole-slide images (WSIs) with generated IMC multiplex, HistoPlexer improves both survival and immune subtype prediction on the TCGA–SKCM dataset, indicating its potential for enhancing clinical decision-making.

## Results

### Histopathology-based protein multiplex generation with HistoPlexer

**Fig. 1:**
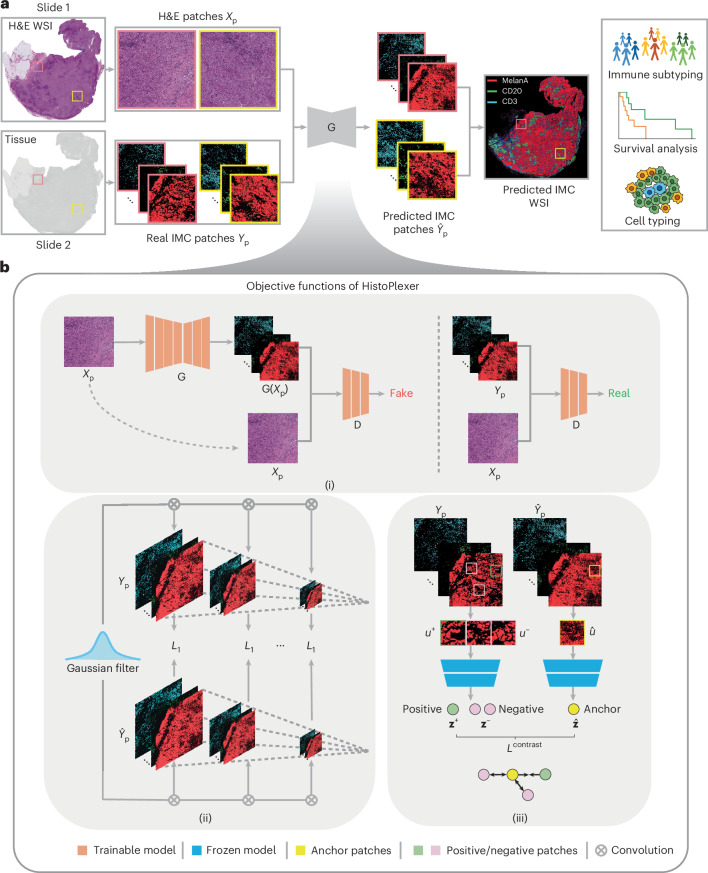
Overview of HistoPlexer architecture. **a**, The HistoPlexer consists of a translator G that takes H&E and IMC images as input and predicts protein multiplexes from morphology information encoded in the H&E images, ultimately generating protein multiplex on the WSI level from H&E input. **b**, The objective functions of HistoPlexer contain the GAN adversarial loss (i), Gaussian pyramid loss with average *L*_1_ distance across scales (ii), and patch-wise contrastive loss with anchor from generated IMC and positive and negative from GT IMC (iii).

HistoPlexer is a generative model based on a conditional generative adversarial network (cGAN) that predicts spatially resolved profiles of multiple proteins from input H&E images. The model is trained on paired H&E and multiplexed IMC images (Fig. 1a) extracted from aligned H&E and IMC ROIs. During training, the H&E images are input to the translator G, which learns to generate protein multiplexes (that is, IMC images) based on the tissue morphology from high-resolution H&E images. The discriminator D receives the generated IMC and input H&E images and scores their similarity to ground-truth (GT) IMC images (Fig. 1b (i)). The translator and discriminator are trained adversarially using a least-squares GAN loss, such that the generated IMC images can deceive the discriminator into classifying them as real. We incorporate two additional losses to ensure both pixel-level and patch-level consistency between the generated and GT IMC images. The pixel-level consistency loss calculates the *L*_1_ distance between the generated and GT IMC images. However, because the H&E and GT IMC images are obtained from serial sections of the tissue block, a degree of spatial displacement in tissue organization exists between consecutive slices (termed slice-to-slice variation). Although aligned at the structural level via template matching, consecutive slides from real-world diagnostic material are not pixel-level aligned. To account for these differences, we adopt the Gaussian pyramid loss^12^, which relaxes the alignment constraint by evaluating the similarity between the generated and GT IMC images at multiple scales (Fig. 1b (ii)). For patch-level consistency, we utilize a patch-wise contrastive loss to ensure that corresponding patches in the generated and GT IMC images are closer in the embedding space than distant ones (Fig. 1b (iii)). We also incorporate adaptive weights for different patches based on their proximity to GT, as described in ref. ^21^.

We built the HistoPlexer framework using a multimodal metastatic melanoma dataset generated by TuPro^18^. Each patient was characterized by multiple modalities, including H&E and IMC images. ROIs of 1 mm^2^ were selected on each H&E WSI based on visual inspection by a pathology expert, and IMC data were generated for those ROIs on a consecutive section of the same tumour block. Using template matching^22^, we created a paired dataset of 336 H&E and IMC ROIs from 78 patients. We focused on predicting 11 protein markers that are essential for characterizing the tumour and its surrounding TME. These included tumour markers (MelanA, S100, gp100, SOX10), immune markers (CD3, CD8a, CD20, CD16, CD31) and antigen-presentation markers (HLA-ABC, HLA-DR).

### HistoPlexer generates accurate and realistic protein multiplexes

We compared HistoPlexer with Pix2pix^23^ and PyramidP2P^12^ in two settings: multiplex (MP), where a single model predicts all markers simultaneously, and singleplex (SP), where separate models predict each marker individually, with predictions stacked to form a pseudomultiplexed output. All models were trained on 231 ROIs and tested on 105 ROIs.

**Fig. 2:**
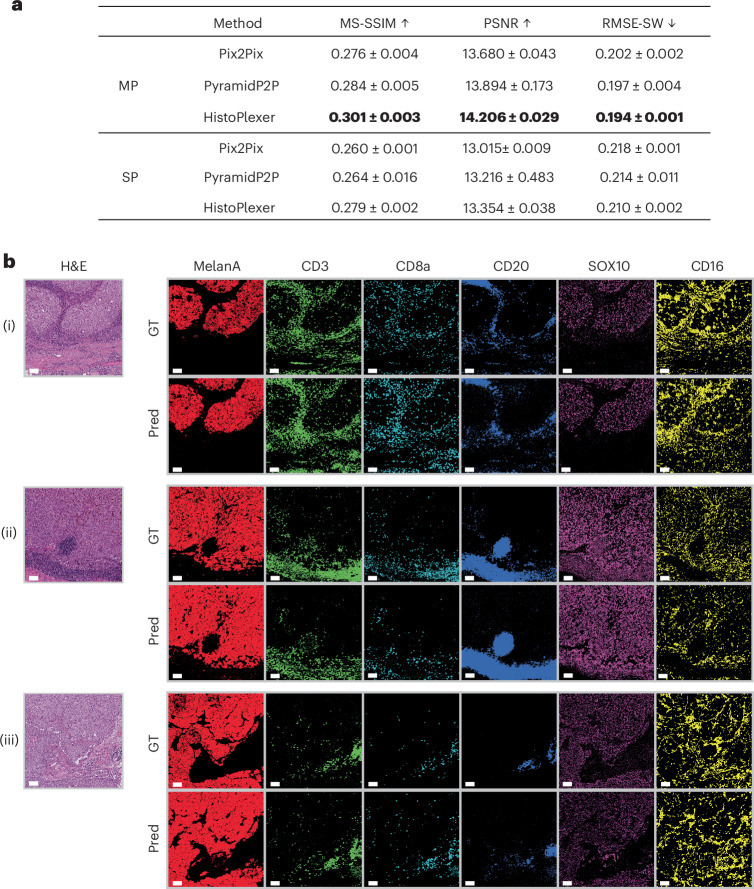
ROI-level assessment of HistoPlexer. **a**, Quantitative assessment and comparison against benchmarks using MS-SSIM, PSNR and RMSE-SW for MP and SP settings. The up arrow indicates higher values are better. The down arrow indicates lower values are better. The best results are highlighted in bold. **b**, Qualitative assessment of HistoPlexer for three ROIs ((i)–(iii)) with H&E as input to HistoPlexer, and expression profiles of individual markers: MelanA, CD3, CD8a, CD20, SOX10 and CD16. Top rows: GT expression profiles from IMC modality; bottom rows: predicted (Pred) expression profiles from HistoPlexer. Scale bars, 100 μm. Source data

**Extended Data Fig. 1:**
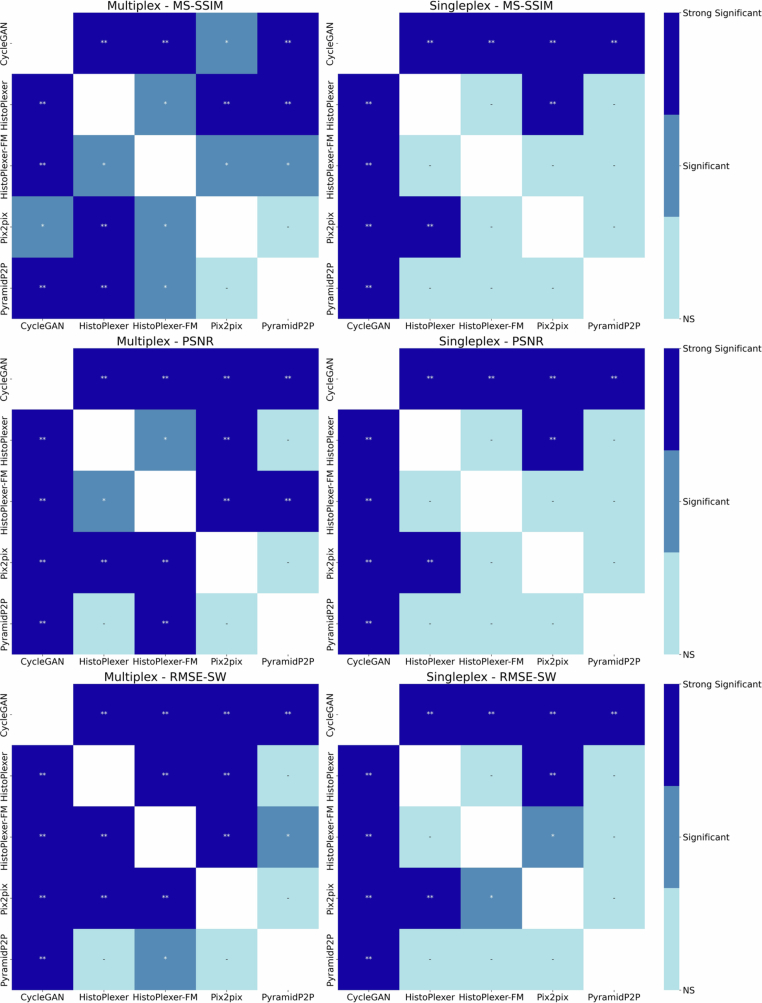
Statistical test for HistoPlexer and baselines. Paired two-sided t-test: Comparison of significance levels between methods in multiplex (left) and singleplex (right) settings for MS-SSIM (row 1), PSNR (row 2) and RMSE-SW (row 3) metrics. No adjustments were made for multiple comparisons. Significance levels are colour-coded: Not Significant (NS) (p≥0.05), Significant (p<0.05), and Strong Significant (p<0.01).

We assessed generated IMC image quality using the multiscale structural similarity index (MS-SSIM)^24^ for perceptual similarity across multiple scales, root mean squared error using sliding window (RMSE-SW)^25^ for pixel-level differences and peak signal-to-noise ratio (PSNR)^26^ for signal fidelity. HistoPlexer in the MP setting achieves the highest MS-SSIM and PSNR and lowest RMSE-SW (Fig. 2a), indicating greater similarity to GT IMC images generated from consecutive tissue sections. The MP setting consistently outperforms SP across all methods, suggesting simultaneous marker prediction captures intermarker correlations. For completeness, we included a comparison with CycleGAN^27^ (Extended Data Table 1; statistical test results shown in Extended Data Fig. 1) and reported the performance of individual markers for the HistoPlexer-MP model (Extended Data Table 2). Moreover, HistoPlexer surpasses baseline methods on pixel-aligned DeepLIIF datasets^14,20^, as shown in Extended Data Tables 3 and 4, with details in Supplementary Information.

**Extended Data Fig. 2:**
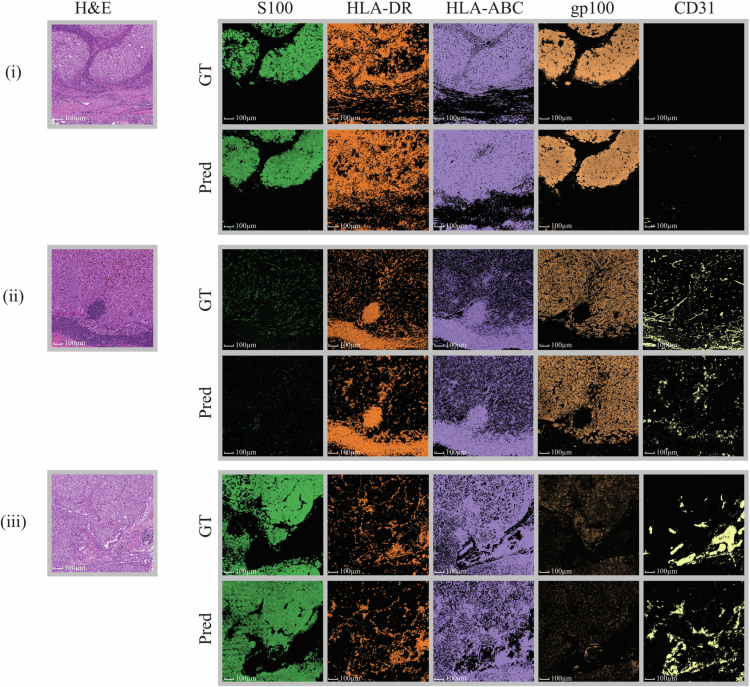
Qualitative RoI-level assessment of HistoPlexer. H&E (first column) and expression profiles of individual markers: S100, HLA-DR, HLA-ABC, gp100 and CD31 (from second to last column). Top row: ground-truth (GT) expression profiles; bottom row: predicted (pred) expression profiles. Source data

We qualitatively evaluated the generated IMC images by comparing them with the GT (Fig. 2b and Extended Data Fig. 2) and observed strong alignment in global patterns. However, pixel-level correspondence was not expected due to the inherent slice-to-slice variations. In some instances, we observed confusion between CD20 and the CD3/CD8a markers. For instance, in Fig. 2b (ii), CD20 is overexpressed, whereas CD3 and CD8a are underexpressed. This may result from the visually similar morphology of B and T cells in H&E images, leading to confusion between their markers (CD20 for B cells and CD3/CD8a for T cells)^28^.

To evaluate the perceived realism of generated IMC images, we used the Human Eye Perceptual Evaluation (HYPE) framework^29^, in which experts assess real versus generated IMC images for specific markers shown alongside corresponding H&E images. We construct two evaluation sets: tumour-associated markers (MelanA, S100, gp100, SOX10) and lymphocyte markers (CD20, CD3, CD8a). For each set, two experts independently reviewed 250 image pairs, evenly split between real and generated, sampled from test ROIs and augmented through small translations and rotations. The evaluation yielded mean HYPE scores of 41.8% (±0.3%) for lymphocyte markers and 42.8% (±0.6%) for tumour markers. The generated images achieved HYPE scores of 61.6% (±1.3%) and 72.8% (±1.1%), indicating that the majority (>50%) were perceived as real by domain experts.

**Fig. 3:**
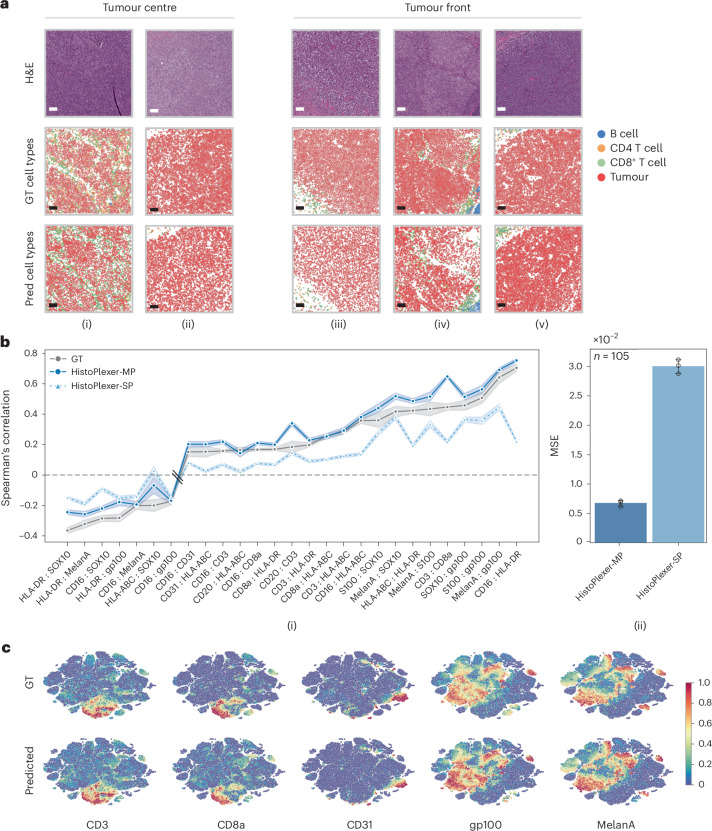
Assessing performance beyond pixel level using cell-type and spatial analyses. **a**, Cell-typing results: ROIs from the tumour centre ((i) and (ii)) and tumour front ((iii)–(v)), showing H&E, GT and predicted cell types in ROIs grouped by their location within the tissue: tumour centre and tumour front. Scale bars, 100 μm. **b**, Spearman’s correlation coefficients between protein pairs, comparing the GT with both SP and MP predictions of HistoPlexer (i). The pairs on the *x* axis are ordered by increasing Spearman’s correlation in the GT. MSE between the GT and predicted Spearman’s correlation coefficients, comparing the SP and MP predictions of HistoPlexer (ii). Bars represent mean values, and error bars indicate standard deviation (s.d.). **c**, Joint *t*-SNE visualization of protein co-localization patterns for selected markers: CD3, CD8a, CD31, gp100 and MelanA. The colour represents normalized protein expression. Source data

Next, we went beyond pixel-level evaluation by identifying relevant cell types. We used GT cell-type annotations from the GT IMC training set, following ref. ^8^, and trained a random forest classifier^30^ based on average marker expression per cell to classify them into five classes: tumour cells, B cells, CD8^+^ T cells, CD4^+^ T cells and others. This classifier was then applied to both GT and generated IMC images in the test set to obtain cell-type maps (Fig. 3a). We visualized ROIs from the tumour centre and the tumour front at the tumour–TME interface and examined spatial patterns based on immune subtype labels. We observed that immune ‘hot’ tumours, characterized by high immune cell infiltration, showed strong interactions between tumour and CD8^+^ T cells (Fig. 3a (i)), whereas immune ‘cold’ tumours, with low immune presence, displayed minimal immune cell interaction, especially in the tumour centre (Fig. 3a (ii)). Immune ‘cold’ ROIs at the tumour front likewise exhibited sparse or clustered immune cells with minimal interaction with tumour cells (Fig. 3a (iii)–(v)). The strong alignment between predicted and GT cell-type maps suggests that HistoPlexer effectively captures morphological features in H&E images that are relevant for predicting cell types.

### HistoPlexer preserves spatial co-localization patterns

Given the importance of spatial patterns, as highlighted by refs. ^31,32^, we assess spatial co-localization by quantifying the correlations between protein markers that are simultaneously expressed within a given region. For each protein pair, we compute the Spearman’s correlation coefficient (SCC) between the two proteins and average it across ROIs, considering only those pairs with strong positive (>0.15) or strong negative (<−0.15) correlation in GT IMC images. We then compare the SCC between GT and generated IMC multiplexes.

**Extended Data Fig. 3:**
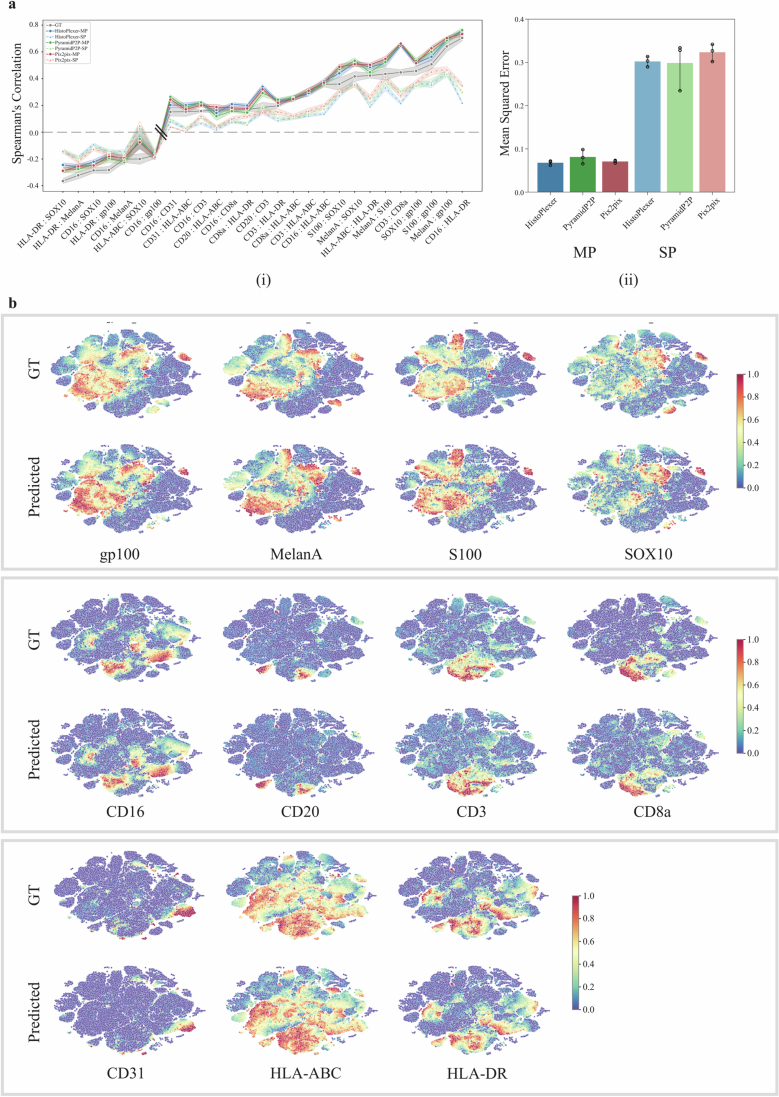
Co-localization patterns. **a**(i) Spearman’s correlation coefficients between protein pairs, comparing the ground truth (GT) with both singleplexed (SP) and multiplexed (MP) predictions of HistoPlexer. **a**(ii) Mean squared error between the GT and predicted Spearman’s correlation coefficients, comparing the SP and MP predictions of HistoPlexer. Bars represent mean values and error bars indicate standard deviation (SD). **b** Joint t-SNE visualization of protein co-localization patterns.

The MP model’s predictions match GT SCC better than the SP model, especially for sparse CD-based immune marker pairs like CD16:HLA-DR, CD3:HLA-ABC and CD16:CD8a, which are underrepresented in the training data (Fig. 3b (i)). We hypothesize that sparse markers lack sufficient tissue context for the SP model to generate accurate predictions. In contrast, the MP model benefits from learning intermarker correlations by predicting all markers simultaneously and leveraging auxiliary morphological information from abundant markers. However, for some protein pairs (for example, CD3:CD8a and CD20:CD3), the SCC in MP exceeds that of the GT. This likely stems from similar morphologies of CD8^+^ T cells, CD3 T cells and B cells (CD20) in H&E images^28^, potentially leading to overprediction of these sparse markers and overestimated co-localization patterns. Additionally, we compute the mean square error (MSE) between SCC values of GT and generated IMC across test ROIs (Fig. 3b (ii)). The MP model achieves an MSE approximately an order of magnitude lower than that of the SP model, supporting our hypothesis. Comparisons with Pix2Pix^23^ and PyramidP2P^12^ are in Extended Data Fig. 3a. See Supplementary Information for biological implications of observed patterns.

To explore spatial patterns beyond protein pairs, we visualized expression profiles using *t*-distributed stochastic neighbour embedding (*t*-SNE) of cells from both GT and generated IMC multiplexes, following ref. ^33^. We observed a strong correspondence between *t*-SNE embeddings from both GT and generated IMC multiplexes (Fig. 3c). For instance, cells that are positive for CD3 and CD8a are concurrently negative for CD31, gp100 and MelanA. This aligns with biological expectations, as CD3 and CD8a are expressed on T cells but not on endothelial (CD31) or tumour (gp100, MelanA) cells. Full *t*-SNE plots for all markers are provided in Extended Data Fig. 3b.

In conclusion, our quantitative and qualitative results show that the spatial co-localization patterns in GT can be effectively replicated using the generated IMC images. These patterns remain consistent across tissue sections, providing a robust evaluation metric that mitigates slice-to-slice variations.

### HistoPlexer enables multiplexed proteomics profiling on the WSI level

**Fig. 4:**
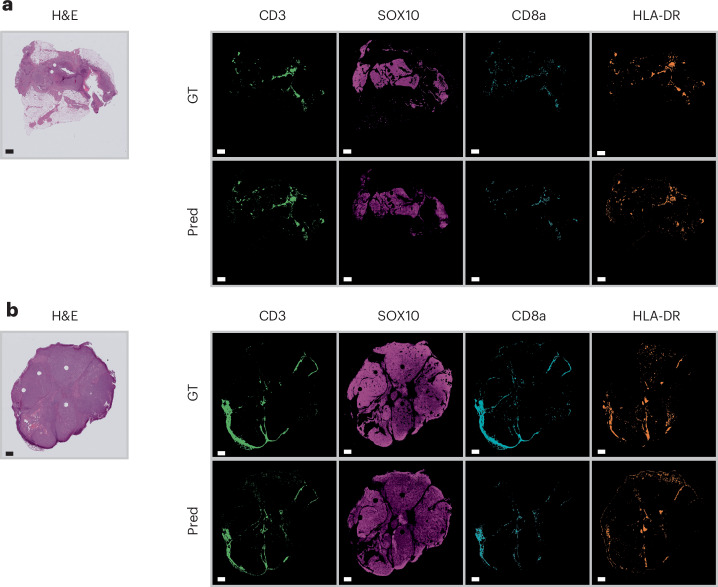
Qualitative WSI-level assessment of HistoPlexer. **a**,**b**, H&E staining (first column; **a** and **b**) and expression profiles of individual markers (CD3, SOX10, CD8a and HLA-DR, from second to last column; **a** and **b**). Top rows: GT expression profiles from Ultivue images; bottom rows: Pred expression profiles at the WSI level for samples in **a** and **b**. Scale bars, 1 mm.

HistoPlexer enables the generation of IMC images from H&E WSIs of up to 100,000 × 100,000 pixels, allowing for the simultaneous visualization of multiple protein markers across entire tissue sections. This capability offers a comprehensive view of tumour and TME interactions at the WSI level. Because GT IMC data are available only for ROIs, we use Ultivue’s InSituPlex technology to obtain multiplexed WSIs using the Immuno8 and MDSC FixVue panels. These panels include key markers, such as SOX10 for tumours, HLA-DR for antigen presentation and CD3/CD8a for T cell profiling, which are shared with the generated protein multiplex. Figure 4 provides a qualitative comparison between the generated IMC and Ultivue multiplex at the WSI level. In both cases, a high concordance in global structures and hotspot regions is observed across all markers. In Fig. 4b, whereas there is good alignment for CD3 and SOX10 markers, differences are observed for CD8A and HLA-DR, particularly at the tissue edges (for example, the bottom-left border). These differences are likely due to slice-to-slice variations between H&E and Ultivue images, which lead to slight shifts in tissue boundaries.

### HistoPlexer facilitates immune phenotyping

**Fig. 5:**
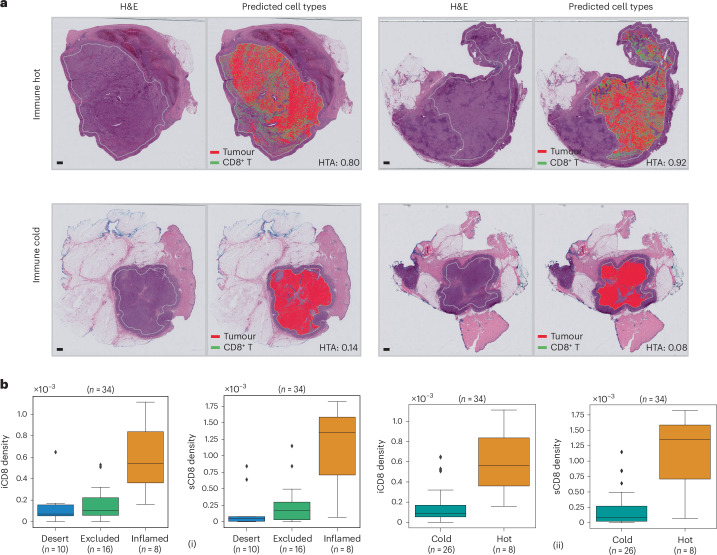
Immune phenotyping using HistoPlexer. **a**, Four samples from TuPro metastatic melanoma cohort with two immune-hot cases in the top row and two immune-cold samples in the bottom row. For each sample, H&E image on the left along with overlay of predicted tumour and CD8^+^ T cells within tumour centre region using HistoPlexer model on the right. Heterogeneity average (HTA) index for quantifying the spatial heterogeneity or interaction between tumour and CD8⁺ T cells is indicated for each sample. Scale bars, 1 mm. **b**, Box plot of iCD8 and sCD8 CD8^+^ T cell densities in tumour centre compartment, stratified by immune desert, excluded and inflamed classes (i). Box plot of iCD8 and sCD8 CD8^+^ T cell densities in tumour centre compartment, stratified by immune-hot and -cold classes (ii). Box plots show the median (centre line), the 25th and 75th percentiles (box limits), and whiskers extending to the most extreme data points within 1.5× the interquartile range from the box limits. Points beyond the whiskers represent outliers.

We demonstrated the utility of HistoPlexer for immune phenotyping by stratifying immune subtypes based on the spatial distribution of CD8^+^ T cells, using only H&E images from TuPro metastatic melanoma samples. As illustrated in Fig. 5a, we visualized predicted tumour cells and CD8^+^ T cells across H&E WSIs. In immune-hot cases, characterized by substantial CD8^+^ T cell infiltration and typically associated with better response to immunotherapy^34,35^, we observed both tumour (attacker) cells and infiltrating CD8^+^ (defender) T cells within the tumour region, suggesting an active immune response. In contrast, immune-cold cases exhibit minimal or no CD8^+^ T cell infiltration in the tumour area, correlating with poor immunotherapy outcomes. Building on the immune subtype classification framework proposed in ref. ^5^, we further quantified intratumoral (iCD8) and stromal (sCD8) CD8^+^ T cell densities within the tumour centre compartment. To do this, we first localized CD8^+^ T cells using HistoPlexer and then annotated and segmented the tumour centre into intratumoral and stromal regions using the HALO^AI^ platform across 34 TuPro melanoma samples. Figure 5b (i) shows immune subtype stratification using iCD8 and sCD8 densities (measured per μm^2^). We observe that immune desert cases exhibit very low iCD8 and sCD8 density, indicating the presence of only rare or isolated CD8^+^ T cells. Immune excluded cases also show very low iCD8 density but slightly higher sCD8 density compared to immune desert cases, suggesting some CD8^+^ T cells have reached the stroma but not the intratumoral regions. Inflamed cases display high densities of both iCD8 and sCD8, indicating the presence of CD8^+^ T cells in the stromal compartment and, most importantly, their infiltration into intratumoral regions. These trends are consistent with prior findings^5^, validating the utility of our approach. To assess the clinical relevance, we analysed the distribution of iCD8 and sCD8 densities across immune-hot (inflamed) versus immune-cold (excluded and desert) cases (Fig. 5b (ii)). As expected, immune-hot cases exhibit substantially higher densities of both iCD8 and sCD8 compared to immune-cold cases. Furthermore, a random forest classifier trained to distinguish between immune-hot and immune-cold cases achieves an *F*1-score of 0.873 (s.d. 0.006) and a macro-average area under the receiver operating characteristic curve of 0.845 (s.d. 0.047) under 5-fold cross-validation. In conclusion, we demonstrate that HistoPlexer can accurately support immune phenotyping directly from H&E images. This capability offers potential utility in treatment decision-making and patient stratification. For clinical implications of immune phenotyping, refer to Supplementary Information.

### HistoPlexer generalizes to an independent patient cohort

**Fig. 6:**
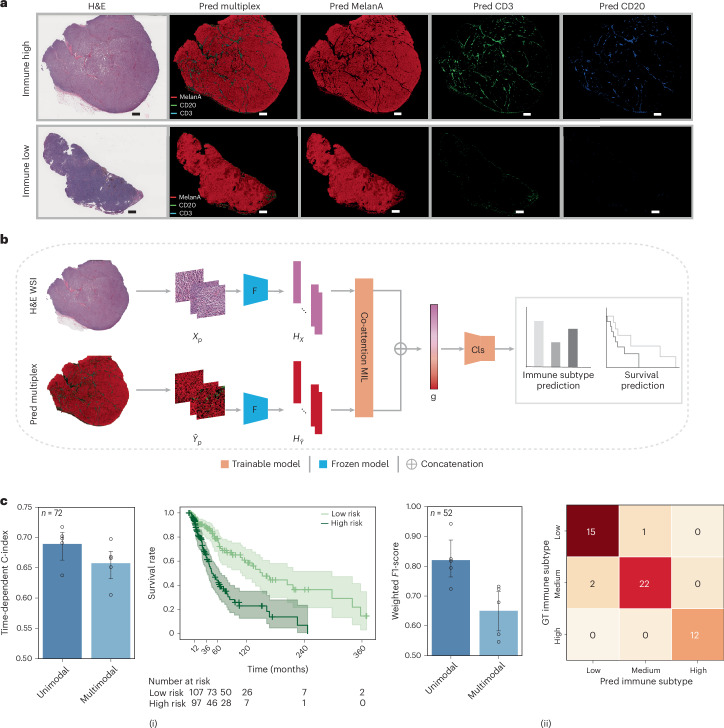
Generalization to an independent patient cohort. **a**, Two examples (immune-high and -low) from the TCGA–SKCM cohort, showing H&E images, predicted protein multiplexes and expression profiles of MelanA, CD3 and CD20 markers. Scale bars, 2 mm (top) and 2.5 mm (bottom). **b**, Model architecture for multimodal survival and immune subtype prediction. **c**, Survival prediction results (i), displaying time-dependent C-index scores (left) and Kaplan–Meier survival curves for all test patients aggregated across five cross-validation folds for the multimodal setting, with separation of low- and high-risk groups (right). Immune subtype prediction results (ii), showing the weighted *F*1-score (left) and confusion matrix (right) for classification into low, intermediate and high immune subtypes. The confusion matrix corresponds to the fold with the highest weighted *F*1-score. For bar plots, bars represent mean values and error bars indicate s.d. Cls, classifier head; F, residual neural network feature extractor ResNet18; *H*_*X*_, H&E IMC features; *H*_*Ŷ*_, predicted IMC features. Source data

We evaluated the generalizability of the HistoPlexer on independent test data from the TCGA–SKCM cohort^19^. Figure 6a shows the generated protein multiplex at the WSI level, along with expression profiles for three markers: tumour-associated MelanA, T cell marker CD3 and B cell marker CD20. In the immune-high sample, we observe higher expression and tumour infiltration of CD3 and CD20 markers, contrasting with the minimal or absent expression in the immune-low case, where immune labels are based on bulk RNA sequencing data^36^.

To explore the clinical utility of the generated IMC images, we assessed their value in augmenting outcome prediction tasks using the MelanA, CD3 and CD20 expression profiles, which are known to have prognostic relevance^37,38^. We extracted features from both the H&E and generated IMC WSIs using pretrained encoders and input them to an attention-based multiple instance learning (MIL) predictor^39^. We trained the MIL predictor under two settings: (1) the unimodal setting, where only H&E features were input to the predictor and (2) the multimodal setting, where features extracted from the corresponding H&E and predicted IMC patches were first aggregated via a co-attention layer^40^, and the bag-level representations of H&E and predicted IMC WSIs after the MIL pooling layer were concatenated before being fed into the classification head (Fig. 6).

We benchmark the model on two clinically relevant tasks: immune subtype classification and survival prediction. For the survival task, we use disease-specific survival from patient metadata, which offers a more accurate representation of the patient’s disease status^41^. For immune subtyping, we classify patients into low, intermediate and high immune categories based on bulk RNA sequencing–derived labels^36^. Overall, the multimodal setting consistently outperforms the unimodal configuration for both tasks. For survival prediction, incorporating features from generated IMC images yields a 3.18% improvement in average time-dependent C-index^42^ across 5-fold cross-validation. Kaplan–Meier curves generated from the multimodal model show clear stratification of patients into low- and high-risk groups based on predicted risk scores (definition provided in the section ‘MIL-based clinical outcome prediction’), with statistical significance confirmed by a log-rank test (*P* = 5.05 × 10^−7^). For immune subtype classification, combining features from H&E and generated IMC boosted the average weighted *F*1-score by 17.02% over 5-fold cross-validation. In summary, these findings highlight both the generalizability of HistoPlexer to external cohorts and the clinical utility of the generated protein expression maps in supporting downstream predictive modelling and decision support.

## Discussion

In this study, we introduced HistoPlexer, a generative model for predicting high-order (11-channel) multiplexed protein expression profiles, including both tumour and immune markers, directly from H&E images. By simultaneously predicting markers, HistoPlexer effectively captured sparse markers and preserved biologically meaningful relationships, as validated through spatial correlation analysis of protein co-localization patterns. Our comprehensive evaluation showed consistent outperformance of the MP approach over SP alternatives, with higher MS-SSIM and PSNR and lower RMSE-SW and MSE values. Domain experts found the generated IMC images realistic, with HYPE error rates of 61.6% and 72.8% for lymphocyte and tumour markers. To assess the broader applicability of HistoPlexer across different cancer types, we benchmarked it on the DeepLIIF datasets^14,20^, which provide pixel-aligned H&E and multiplexed images for lung and head and neck cancers. In both settings, HistoPlexer consistently outperformed baseline methods, indicating robustness across diverse tissue types and imaging conditions (Extended Data Tables 3 and 4).

We further explored integrating features from the UNI computational pathology foundation model^43^ to enhance the performance of HistoPlexer, marking one of the first attempts to apply pathology foundation models to generative tasks. Surprisingly, end-to-end training of HistoPlexer without UNI-derived features resulted in lower MSE and better preserved biological relationships (Supplementary Information and Extended Data Table 1). This aligns with recent findings^44^, highlighting the importance of task-specific training for complex biological tasks. Foundation models, typically designed as encoder-only architectures, excel in discriminative tasks like classification and regression by focusing on global high-level features. However, they are less suited for generative tasks that require detailed spatial relationships and fine-grained image details essential for accurate image generation.

We demonstrated clinical utility through two applications. First, HistoPlexer enabled immune phenotyping at the WSI level by quantifying CD8^+^ T cell infiltration patterns (iCD8, sCD8) in tumour centres. These patterns aligned with a state-of-the-art approach^5^ and effectively stratified patients into immune-hot and -cold subtypes, relevant for immunotherapy decisions. Second, we showed generalizability to an independent cohort (TCGA–SKCM) where HistoPlexer-generated features, when used alongside H&E features, improved predictive performance in survival analysis (3.18% increase in time-dependent C-index) and immune subtype classification (17.02% improvement in weighted *F*1-score). These results highlight the potential of HistoPlexer to augment clinical decision-making.

Our study has some limitations. First, in some cases, the model confused CD3/CD8a T cell and CD20 B cell markers, which share morphological similarities. Although this did not substantially impact downstream tasks, it is a priority for future work to refine the model’s ability to accurately distinguish between finer cell types. Second, our focus was on major immune and tumour cell types, such as CD8^+^ T cells, CD4^+^ T cells, B cells and tumour cells. This set could be further extended to include more sparse cell types, such as endothelial cells, by obtaining a larger training cohort. Third, for the multimodal learning experiments on TCGA–SKCM, we limited marker selection to MelanA, CD3 and CD20 based on clinical relevance. Extending the marker panel could further improve downstream applications. Finally, due to inherent slice-to-slice variations in the data, we prioritized downstream task performance over strict pixel-level accuracy.

Our work opens several promising research directions. First, expanding HistoPlexer to additional protein markers and cancer types presents an opportunity to uncover deeper insights into disease mechanisms without requiring additional tissue or increasing the cost. Achieving broader generalization, including the development of a universal model across cancer types, remains a key challenge that will require access to larger, more diverse and harmonized datasets. Our current results serve as a proof of concept and provide a foundation for such future efforts. Second, by releasing the Ultivue InSituPlex dataset generated in this study, we encourage researchers to explore new diffusion models for multiplexed protein marker generation, particularly those that account for slice-to-slice variations. Finally, integrating predicted protein expression maps with other modalities could deepen our understanding of tumour biology and improve patient stratification.

In summary, HistoPlexer advances computational pathology by enabling efficient and cost-effective generation of multiplexed protein profiles from standard histology slides. Our findings demonstrate its potential for clinical integration and its utility in enhancing cancer diagnostics and personalized treatment strategies.

## Methods

### Datasets and preprocessing

#### Tumour Profiler dataset

We built our HistoPlexer framework using a subset of highly multimodal metastatic melanoma dataset generated by TuPro^18^. Each patient was characterized using multiple technologies, including Digital Pathology and IMC. A total of six ROIs of 1 mm^2^ were selected on each H&E WSI, three within the tumour centre and three at the tumour front (intersection of tumour and TME). IMC data were generated for those six ROIs on a consecutive section of the same tumour block. The IMC data were generated at a resolution of 1 μm per pixel, and H&E images were scanned at a resolution of 0.25 μm per pixel. Therefore, ROIs of 1 mm^2^ were represented by 1,000 pixels for IMC data and 4,000 pixels for H&E images. Because the paired data were generated by visually choosing ROIs, in many cases a considerable positional shift and rotation between the specified H&E regions and the resulting IMC regions can be observed. This was overcome by using template matching^45^. Specifically, for the IMC modality, we utilized the tumour channel marker SOX10, which provided valuable structural information of the tissue. For the H&E modality, we focused on the H channel, which captures the haematoxylin staining essential for visualizing cellular structures. Given that H&E and IMC represent different modalities, we employed mutual information as a metric for template matching. This approach allowed us to quantify the alignment between the two datasets, ensuring a robust pairing process. This resulted in a paired dataset of 336 H&E and IMC ROIs from 78 patients for training and testing model performance.

IMC profiling was performed using a panel of 40 antibodies, of which 11 were selected for this study based on the biological function of the corresponding proteins as well as high SNR. The proteins targeted by the 11 antibodies include cell-type markers, such as tumour markers (MelanA, gp100, S100, SOX10), lymphocyte markers (CD20, CD16, CD3, CD8a) and an endothelial marker (CD31). Moreover, two functional markers corresponding to proteins involved in antigen presentation (HLA-ABC, HLA-DR) were included in the protein set.

The raw IMC images were processed with CellProfiler software for cell segmentation^46^. The protein counts extracted from the images were first clipped to 99.9% per protein to exclude outliers and then transformed using the arcsinh-function with cofactor one^47^. To exclude background noise, we applied Otsu thresholding^48^ with kernel size three and sigma three and the threshold, separating signal from background, determined per sample using all available ROIs. The resulting data per protein was first centred and standardized and then subjected to min–max transformation, all using data statistics based on the train set only.

The data were split at the patient level into train and test sets, stratified by immune phenotype (inflamed, immune excluded and immune desert). The stratification ensured the representation of both tumour and immune cells in each set. The patient-level splitting guaranteed that all ROIs from a given patient belonged to only one set, preventing undesired information flow. The resulting train and test sets consisted of 231 and 105 ROIs, respectively. During model training, ROIs were chosen at random, and a tile of size 1,024 × 1,024 from the H&E image and a corresponding IMC region of 256 × 256 was extracted.

For WSI inference, we developed a modular pipeline that processes WSIs in a tile-based approach to create spatially resolved protein multiplexes. The process begins with tissue segmentation, which identifies stained tissue areas and removes the background using Otsu thresholding^48^. Each segmented tissue region is then divided into 1,024 × 1,024 pixel tiles. To optimize performance, we employ a dataloader that batches and parallelizes the processing of these tiles instead of processing them sequentially. We also utilize the Shapely library^49^ for geometric operations, such as creating polygons for tissue masks, scaling them to match the WSI resolution and efficiently determining overlaps between tiles and tissue regions. This approach reduces computational load by concentrating only on relevant areas of the WSI and avoiding background processing. Each tile may undergo optional stain normalization using the Macenko method^50^ to reduce staining variability and ensure colour consistency across images. The tiles are then processed through the trained HistoPlexer model to predict protein multiplexes. After prediction, the generated tiles are stitched together to reconstruct the full WSI at multiple resolutions. We also generate quality-control images to visualize the tiling and segmentation results, and we save the coordinates of the tiles for reproducibility. For visualization, we apply min–max normalization using the min–max values from the training set for all markers.

#### Ultivue dataset

For qualitative evaluation of HistoPlexer on WSIs, we employed Ultivue InSituPlex technology to obtain multiplexed images using the Immuno8 and MDSC FixVue panels. The Immuno8 panel focuses on immune landscape characterization with markers such as CD3, CD4, CD8, CD68, PD-1, PD-L1, FoxP3 and PanCK/SOX10. The MDSC panel identifies myeloid-derived suppressor cells using markers CD11b, CD14, CD15 and HLA-DR. Ultivue images were acquired at a resolution of 0.325 μm per pixel. For evaluation, we used CD3, SOX10, CD8a and HLA-DR markers to assess visual similarity between the generated protein multiplex and Ultivue images.

Paired H&E and Ultivue WSIs were generated by first staining H&E on one tissue section, followed by acquiring Immuno8 and MDSC data on consecutive sections for ten samples. A tonsil tissue was included with each sample as a positive control. Image registration between H&E and Ultivue WSIs was performed using an unsupervised multimodal method^51^, leveraging the DAPI nuclear stain in Ultivue for alignment with H&E images. Both Ultivue and generated IMC images underwent min–max normalization and histogram equalization. Additionally, adaptive thresholding was applied to Ultivue images to reduce noise and extract true signals. Regions with false signals, particularly those corresponding to haemorrhage, bleeding or erythrocytes in H&E, were manually annotated and excluded from the analysis. The dataset, including H&E and Ultivue images, alignment matrices, and annotated excluded regions, is publicly available and can be accessed on Hugging Face (https://huggingface.co/datasets/CTPLab-DBE-UniBas/HistoPlexer-Ultivue). The dataset could serve as a valuable baseline for the field.

#### TCGA–SKCM

Diagnostic WSIs of SKCM were downloaded from the TCGA database (https://portal.gdc.cancer.gov/) for a total of 472 cases. Clinical data of SKCM samples including age, gender, sample type (primary tumour/metastatic) and disease-specific survival were also downloaded. For the survival prediction, we discarded cases where the diagnostic WSIs were of low resolution or the disease-specific survival data were missing, leaving 360 cases in total. For the immune subtype prediction, we kept a total of 257 cases where immune subtype labels were available. For each task, we randomly split the cases stratified by age, gender and sample type to create 5-fold cross-validation with a 4:1 ratio of training-validation sets.

### HistoPlexer architecture

The HistoPlexer is based on cGAN, which takes an H&E image as input condition and generates multiplexed IMC images where each corresponds to a spatially resolved protein expression profile. The translator of the HistoPlexer is a fully convolutional U-Net^52^ consisting of an encoder and a decoder. The encoder comprises six downsampling blocks, each with a convolution layer of stride 2 and kernel size 3. The decoder comprises five upsampling blocks, each with nearest-neighbour interpolation, followed by a convolution layer of stride 1 and kernel size 3. Each layer is followed by a batch-norm layer and ReLU activation. The discriminator consists of six blocks, each with a convolution layer followed by a spectral normalization layer and ReLU activation. We used patches extracted from template-matched pairs of H&E and IMC ROIs to train the HistoPlexer and optimize the model with three objectives: an adversarial loss to enforce image-level consistency, a Gaussian pyramid loss to enforce pixel-level consistency and a patch-wise contrastive loss to enforce patch-level consistency.

#### Adversarial loss

We used the least-squares loss proposed in LSGAN^53^ as our adversarial loss and the binary coding scheme, where 0 and 1 are the labels for generated (that is, fake) and real IMC images, respectively. We also adopt the multiscale gradient approach^54^, which allows simultaneous gradient propagation at multiple scales (that is, resolutions). Considering a set of scales {*s* ∈ *S*}, the multiscale adversarial losses for the translator G and discriminator D are formulated as

1 $$\begin{array}{rcl}{L}_{\rm{G}}^{\,\text{adv}\,}&=&\frac{1}{| S| }{{\mathbb{E}}}_{{{x}}_{\rm{p}} \sim {X}_{\rm{p}}}\left[{\left({D}({{G}}^{(s)}({{x}}_{\rm{p}})| {{x}}_{\rm{p}})-1\right)}^{2}\right],\\ {L}_{\rm{D}}^{\,\text{adv}\,}&=&\frac{1}{| S| }\sum _{s\in S}\left[{{\mathbb{E}}}_{\begin{array}{c}{{x}}_{{\rm{p}}} \sim {X}_{{\rm{p}}}\\ {{y}}_{{\rm{p}}} \sim {Y}_{{\rm{p}}}\end{array}}\left[{({D}({{y}}_{{\rm{p}}}| {{x}}_{{\rm{p}}})-1)}^{2}\right]+{{\mathbb{E}}}_{{{x}}_{{\rm{p}}} \sim {X}_{{\rm{p}}}}\left[{({D}({{G}}^{(s)}({{x}}_{{\rm{p}}})| {{x}}_{{\rm{p}}}))}^{2}\right]\right].\end{array}$$

where *X*_p_ = {*x*_p_ ∈ *X*_ROI_} and *Y*_p_ = {*y*_p_ ∈ *Y*_ROI_} denote paired training patches sampled from template-matched H&E and IMC ROIs, respectively; *G*^(*s*)^(⋅) and *D*(⋅) denote the mapping functions parameterized by the translator (at the output scale *s*) and discriminator, respectively; and ∣⋅∣ denotes the cardinality of a set.

#### Gaussian pyramid loss

We also implement a pixel-level *L*_1_ loss as in ref. ^23^. Because our H&E and GT IMC images were not pixel-aligned, we relaxed the constraint on pixel-to-pixel correspondence by calculating the *L*_1_ loss at multiresolution representations of the generated and GT IMC images^12^, termed Gaussian pyramid loss^12^. More specifically, a Gaussian pyramid was constructed through iterative Gaussian smoothing and downsampling. Each level of resolution, termed an octave, comprised a series of images with increasing degrees of smoothness. Transition between resolutions was achieved by downsampling the image at the highest smoothness level of the current octave to initiate the next:

$${{y}}_{{\rm{p}},1}^{r+1}=\,{\rm{Downsample}}\,\left({{y}}_{{\rm{p}},\,\#{\rm{gs}}\,}^{r}\right)$$

where *#*gs denotes the number of Gaussian smoothings at one resolution. Note that for the generated IMC images, we only computed the Gaussian pyramid on the final output scale. Considering a set of resolutions {*r* ∈ *R*}, the Gaussian pyramid loss is a weighted sum of *L*_1_ loss computed on the primary layer of each octave, formulated as

2 $${L}^{{\rm{gp}}}=\sum _{r\in R}{w}_{r}{{\mathbb{E}}}_{\begin{array}{c}{{x}}_{{\rm{p}}} \sim {X}_{{\rm{p}}}\\ {{y}}_{{\rm{p}}} \sim {Y}_{{\rm{p}}}\end{array}}{\left\Vert {{y}}_{{\rm{p}},1}^{r}-{\hat{{y}}}_{{\rm{p}},1}^{r}\right\Vert }_{1},$$

where ${\hat{{y}}}_{{\rm{p}}}$ denotes the generated IMC image patches, *w*_*r*_ denotes the resolution level and *w*_*r*_ is the weight of the *L*_1_ loss at that level.

#### Patch-wise contrastive loss

We further incorporated a patch-wise contrastive loss, inspired by ref. ^21^. More specifically, we first extracted multilayer features using a pretrained feature encoder and applied a transformation via a small projection head (for example, a multilayer perceptron) on the extracted features to enrich their expressiveness^55^. Then, we randomly selected a set of pixel locations for each feature layer. By aggregating selected patch features from each layer, we could obtain two feature sets for the generated and GT IMC images, respectively.

Let ${\hat{\bf{z}}}_{l}^{\;i}$ denote the anchor feature of the *i*th patch of the generated IMC image, extracted from the *l*th layer of the feature encoder; ${\bf{z}}_{l}^{i}$ and ${\bar{\bf{z}}}_{l}^{i}$ denote the positive and negative features of the corresponding patch (that is, at the same pixel location) and the collection of non-corresponding patches (that is, at different pixel locations), extracted from the same layer, respectively. Our patch-wise contrastive loss is defined as

3 $${L}^{{\rm{contrast}}}={{\mathbb{E}}}_{\begin{array}{c}{{x}}_{{\rm{p}}} \sim {X}_{{\rm{p}}}\\ {{y}}_{{\rm{p}}} \sim {Y}_{{\rm{p}}}\end{array}}\frac{1}{\#{\rm{layer}}}\frac{1}{\#{\rm{patch}}}\mathop{\sum }\limits_{l=1}^{\#{\rm{layer}}}\mathop{\sum }\limits_{i=1}^{\#{\rm{patch}}}{w}_{t}\left({\hat{\bf{z}}}_{l}^{i},{\bf{z}}_{l}^{i}\right){\ell }_{{\rm{InfoNCE}}}\left({\hat{\bf{z}}}_{l}^{i},{\bf{z}}_{l}^{i},{\bar{\bf{z}}}_{l}^{i}\right),$$

where

$${\ell }_{{\rm{InfoNCE}}}(\bf{z},{\bf{z}}^{+},{\bf{z}}^{-})=-\log \frac{\exp (\bf{z}\cdot {\bf{z}}^{+}/\tau )}{\exp (\bf{z}\cdot {\bf{z}}^{+}/\tau )+\mathop{\sum }\nolimits_{n = 1}^{N}\exp \left.(\bf{z}\cdot {\bf{z}}_{n}^{-})/\tau \right)}$$

is the InfoNCE objective^56^ and

$${w}_{t}({\hat{\bf{z}}}_{l}^{i},{\bf{z}}_{l}^{i})=\left(1-g\left(\frac{t}{T}\right)\right)\times 1.0+g\left(\frac{t}{T}\right)\times h\left({\rm{sim}}\,({\hat{\bf{z}}}_{l}^{i},{\bf{z}}_{l}^{i})\right)$$

is the adaptive patch weight^21^. Here, *#*layer and *#*patch denote the number of layers and patches from which we extract features, *t* and *T* denote the current and total training steps, *h*( ⋅ ) denotes some weighting function and sim( ⋅ ) is some similarity measurement.

Although the HistoPlexer translator outputs the prediction of all selected IMC markers, we encountered a practical limitation when employing a pretrained feature encoder, which often requires an RGB image as input. To circumvent this, we first extracted each channel (that is, marker) of the output IMC image and replicated it along the channel dimension to create a pseudo-RGB image. We then passed each of them to the feature encoder. The final patch-wise contrastive loss is the sum of that of each channel.

The total losses for G and D are formulated as

4 $$\begin{array}{rcl}{L}_{{\rm{G}}}&=&{L}_{{\rm{G}}}^{{\rm{adv}}}+{\lambda }_{{\rm{gp}}}{L}^{{\rm{gp}}}+{\lambda }_{{\rm{contrast}}}{L}^{{\rm{contrast}}}\\ {L}_{{\rm{D}}}&=&{L}_{{\rm{D}}}^{{\rm{adv}}}+{\lambda }_{{R}_{1}}{R}_{1}\end{array}$$

where

$${R}_{1}={{\mathbb{E}}}_{\begin{array}{c}{{x}}_{{\rm{p}}} \sim {X}_{{\rm{p}}}\\ {{y}}_{{\rm{p}}} \sim {Y}_{{\rm{p}}}\end{array}}{\parallel {\nabla }_{{y}}D({{y}}_{{\rm{p}}}| {{x}}_{{\rm{p}}})\parallel }_{2}^{2}$$

is the gradient penalty^57^ and *λ*_gp_, contrast and ${\lambda }_{{R}_{1}}$ are the weights for the Gaussian pyramid loss, patch-wise contrastive loss and gradient penalty, respectively.

#### Implementation and training details

The model was trained for 100 epochs using the ADAM optimizer^58^ with momentum parameters β1 = 0.5 and β2 = 0.999 with learning rates 0.004 and 0.0008 for translator and discriminator networks, respectively. The weights were initialized using Xavier initialization. The batch size was set to 16 and the patch size to 256 for IMC and 1,024 for H&E images, to accommodate for the higher resolution of the latter. We increased the generalization capabilities of the model by adopting data augmentation, including colour augmentation, random flipping, small translations and rotations. We employed the least-squares GAN objective. The weights for loss terms were as follows: *λ*_gp_ = 5.0, *λ*_contrast_ = 1.0 and ${\lambda }_{{R}_{1}}$ = 1.0. For model inference and evaluation, we used the latest checkpoint at the end of model training.

### Evaluation metrics

To evaluate the quality of generated images, we used three widely adopted metrics: PSNR, MS-SSIM and RMSE-SW.

PSNR was used to measure the reconstruction quality by quantifying the ratio between the maximum possible signal power and the power of corrupting noise. It is expressed in decibels, with higher values indicating better image quality. The PSNR is calculated as

5 $${\rm{PSNR}}=10{\log }_{10}\left(\frac{{L}^{2}}{{\rm{MSE}}}\right)$$

where *L* is the dynamic range of the pixel values (for example, 255 for 8-bit images) and MSE represents the MSE between the original image *I* and the generated image *I*′

6 $${\rm{MSE}}=\frac{1}{N}\mathop{\sum }\limits_{i=1}^{N}{\left(I(i)-{I}^{{\prime} }(i)\right)}^{2}$$

MS-SSIM extends the traditional SSIM metric by incorporating multiple scales to capture structural similarity at various resolutions. The SSIM between two images *I* and *I*′ is defined as

7 $${\rm{SSIM}}(I,{I}^{{\prime} })=\frac{(2{\mu }_{I}{\mu }_{{I}^{{\prime} }}+{C}_{1})(2{\sigma }_{I{I}^{{\prime} }}+{C}_{2})}{(\;{\mu }_{I}^{2}+{\mu }_{{I}^{{\prime} }}^{2}+{C}_{1})({\sigma }_{I}^{2}+{\sigma }_{{I}^{{\prime} }}^{2}+{C}_{2})}$$

where *μ*_*I*_ and ${\mu }_{{I}^{{\prime} }}$ are the means, ${\sigma }_{I}^{2}$ and ${\sigma }_{{I}^{{\prime} }}^{2}$ are the variances, and ${\sigma }_{I{I}^{{\prime} }}$ is the covariance between the two images. *C*_1_ and *C*_2_ are small constants to stabilize the division. In MS-SSIM, structural similarity is computed at multiple scales, and the final score is a weighted product of SSIM values across these scales:

8 $${\text{MS-SSIM}}(I,{I}^{{\prime} })=\mathop{\prod }\limits_{j=1}^{M}{\left({{\rm{SSIM}}}_{j}(I,{I}^{{\prime} })\right)}^{{\alpha }_{j}}$$

where *M* is the number of scales and *α*_*j*_ is weighting factor at scale *j*. Higher MS-SSIM values indicate better perceptual similarity.

RMSE-SW is an image quality metric used to assess the similarity between the original image *I* and a generated image *I*′. Unlike traditional RMSE, which computes the error over the entire image globally, RMSE-SW calculates the error within localized regions by moving a fixed-size window across the image. The RMSE-SW between two images *I* and *I*′ is defined as

9 $$\,\text{RMSE-SW}\,=\sqrt{\frac{1}{N}\mathop{\sum }\limits_{k=1}^{N}\frac{1}{w\times h}\mathop{\sum }\limits_{i=0}^{w-1}\mathop{\sum }\limits_{j=0}^{h-1}{\left(I({x}_{k}+i,{y}_{k}+j)-{I}^{{\prime} }({x}_{k}+i,{y}_{k}+j)\right)}^{2}}$$

where *w* × *h* represents the size of the moving window across the image with a defined stride, resulting in *N* windows. For each window, we computed the MSE between the corresponding pixel values of *I* and *I*′.

These metrics provide a comprehensive assessment of both pixel-level accuracy and perceptual similarity of the generated images. Frechet inception distance and kernel inception distance are widely used metrics for evaluating the quality of generated images; however, they are less effective on small datasets as they rely on the mean and covariance of a cohort. Hence they were not used when evaluating HistoPlexer.

To quantify the evaluation by domain experts, we used HYPE score, which measures the error rate at which humans mistake generated images for real ones or vice versa. It is defined as

10 $$\begin{array}{rcl}{\rm{HYPE}}&=&\left(\frac{{\rm{FP}}+{\rm{FN}}}{{\rm{TP}}+{\rm{TN}}+{\rm{FP}}+{\rm{FN}}}\right)\times 100\\ {{\rm{HYPE}}}_{{\rm{fake}}}&=&\left(\frac{{\rm{FP}}}{{\rm{TN}}+{\rm{FP}}}\right)\times 100\\ {{\rm{HYPE}}}_{{\rm{real}}}&=&\left(\frac{{\rm{FN}}}{{\rm{TP}}+{\rm{FN}}}\right)\times 100\end{array}$$

where TP is the number of true positives, TN is the number of true negatives, FP is the number of false positives, and FN is the number of false negatives. HYPE_fake_ and HYPE_real_ are the error rates for generated and real images, respectively.

### HistoPlexer for cell-level analysis

#### Pseudo-cells

Because spatial analyses of IMC data typically rely on cell-level readouts, we created pseudo-single-cell data by extracting circular regions of 10 μm diameter around nuclei coordinates for both input H&E and GT IMC images. Protein expression was averaged across pixels within each pseudo-cell for individual markers. Nuclei coordinates for H&E images were obtained using the HoVer-Net model^28^, and nuclei coordinates and cell-type labels for GT IMC multiplexes were are derived using Ilastik^59^ and CellProfiler^46^, as described in ref. ^8^. For simplicity, we refer to pseudo-cells as ‛cells’ in the following text.

#### Cell typing

We used a random forest classifier^30^ to categorize cells based on the average expression of 11 markers from the HistoPlexer. The classifier distinguished between tumour cells, B cells, CD8^+^ T cells, CD4^+^ T cells and other cells. Training was performed using the scikit-learn library^60^, with hyperparameters (100 base estimators, maximum tree depth of 30) selected based on the lowest out-of-bag error. The model achieved a macro-averaged *F*1-score of 0.81 on an internal test set. We then applied the trained RF classifier to both GT and generated protein expression data to produce cell-type maps for cells in the test set.

#### *t*-SNE on cell-level marker expression

To explore spatial patterns beyond pairwise protein interactions, we conducted a low-dimensional embedding analysis of cell-level marker expression. Following the approach commonly used for mass cytometry data^61^, we subsampled 1,000 cells per ROI from both GT and generated IMCs, resulting in total 2,000 cells per ROI. A joint *t*-SNE dimensionality reduction (two dimensions, perplexity of 50 and 1,000 iterations) was then applied. For visualization, protein abundance was scaled and clipped at the 99th percentile, and the *t*-SNE plots were coloured according to the scaled protein expression^61^.

### Annotations for immune phenotyping

**Extended Data Fig. 4:**
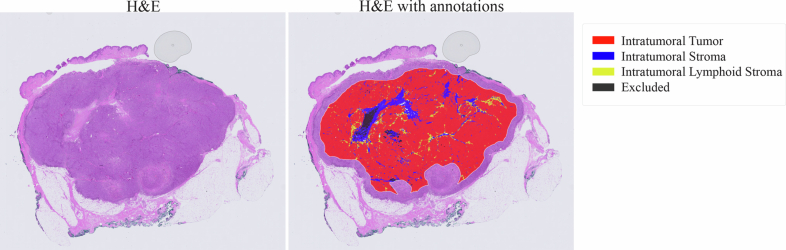
Region annotation for Immune phenotyping. H&E WSI (left) and H&E WSI with an outline of the Tumour Center compartment and annotated regions (right).

To stratify samples into immune subtypes based on the spatial distribution of CD8^+^ T cells, we used annotated regions as established in ref. ^5^. Our dataset included 109 metastatic melanoma H&E WSIs from the TuPro cohort, with metastatic sites in lymph nodes, soft tissue, brain and other distant locations. The primary region for immune-subtyping, termed the ‛tumour centre’, comprised entirely tumour tissue, which was manually defined as a continuous tumour mass excluding a 500 μm margin from the tumour–non-tumour boundary. This ‛tumour centre’ was further segmented into two regions: the ‛intratumoral tumour’ region, consisting of dense clusters of malignant melanocytes without stromal presence, and the ‛intratumoral stromal’ region, which includes extracellular matrix (typically desmoplastic) interwoven within the tumour cell mass but free from malignant melanocytes. These regions were automatically classified using a deep learning model implemented on the HALO^AI^ platform, trained with selected H&E WSI regions. Tissue classification was conducted at 0.30-μm-per-pixel resolution with a minimum object size threshold of 50 μm^2^. Excluded regions—such as preexisting lymphatic tissue, large adipose and muscle regions, artifacts, necrosis, haemorrhage and background—were omitted from the analysis. Ultimately, we analysed 34 samples with the highest-quality tissue classifications from the HALO^AI^ model predictions. Extended Data Fig. 4 shows an example H&E WSI with region annotation and classification.

### MIL-based clinical outcome prediction

#### Attention-based MIL for survival and immune subtype prediction

MIL is a weakly supervised learning method for set-based data structures. In MIL, an input *X* is a bag (that is, permutation-invariant set) of instances *X* = {*x*_1_, …, *x*_*N*_}, where *N* denotes the number of instances in the bag. Given a classification task with *K* classes, the goal is to learn a function ${\mathcal{F}}$ from *M* training pairs ${\{({X}^{(m)},{{y}}^{(m)})\}}_{m = 1}^{M}$ that maps *X* to a bag-level label *y* ∈ *K* without knowing label *y*_*i*_ ∈ *K* for each instance in the bag. In our context, the input is a WSI, and the instances denote the extracted patches. More specifically, we follow the embedding-based MIL approach^39^ and extract a feature vector ${{\bf{h}}}_{i}=h({{x}}_{i})\in {{\mathbb{R}}}^{d}$ from each patch. Then, an attention-pooling operator aggregates the patch features **h**_*i*=1:*N*_ to a single WSI-level representation^39^

$${\bf{g}}=g({{\bf{h}}}_{i})=\mathop{\sum }\limits_{i=1}^{N}{a}_{i}{{\bf{h}}}_{i},$$

where

$${a}_{i}=\frac{\exp \{{{\bf{w}}}^{\top }(\tanh ({V}{{\bf{h}}}_{i})\odot \eta ({U}{{\bf{h}}}_{i}))\}}{\mathop{\sum }\nolimits_{j = 1}^{N}\exp \{{{\bf{w}}}^{\top }(\tanh ({V}{{\bf{h}}}_{j})\odot \eta ({U}{{\bf{h}}}_{j}))\}}$$

is the gated attention^39^. Here, ${\bf{w}}\in {{\mathbb{R}}}^{L},\,{V}\in {{\mathbb{R}}}^{L\times D},\,{U}\in {{\mathbb{R}}}^{L\times D}$ are learnable parameters with hidden dimension *L*, ⊙ is element-wise multiplication, and *η*( ⋅ ) denotes the sigmoid function. Finally, a classifier *f*( ⋅ ) maps the WSI-level representation to a WSI-level label $\hat{{y}}\in K$.

The end-to-end prediction takes the following general form:

11 $$\hat{{y}}={\mathcal{F}}(X\;)=f\left(g\left(\{h({{x}}_{i}):{{x}}_{i}\in X\;\}\right)\right)\,.$$

For survival prediction, we modelled the time-to-event distributions as an ordinal regression task with right censored data (that is, patient death is unobserved until last known follow-up). Following ref. ^40^, we defined discrete time intervals and modelled each interval using an independent neuron in the output layer. More specifically, we partitioned the continuous time scale into non-overlapping time intervals [*t*_*j*−1_, *t*_*j*_), *j* ∈ [1, … , *J*] based on the quartiles of survival time values, denoted as *y*_*j*_. The continuous time-to-event *t*^(*m*)^ for each patient was then replaced by a discrete time label ${{y}}_{j}^{(m)}$, where

$${{y}}_{j}^{(m)}={{y}}_{j}\quad \,\text{if}\,\,{t}^{(m)}\in [{t}_{j-1},{t}_{j})\,\,\text{for}\,\,j\in \{0,\ldots \,J\;\}.$$

The problem then simplified to classification, where each patient defined by a triplet $({{\bf{g}}}^{(m)},{{y}}_{j}^{(m)},{c}^{(m)})$. Here, **g** is the aggregated bag features; *c* is the censorship status, where *c* = 0 if the death of the patient is observed and *c* = 1 otherwise; and *y*_*j*_ is the discrete time GT label. We adopt the negative log-likelihood survival loss^62^ for modal optimization, formulated as

12 $$\begin{array}{ll}{L}_{{\rm{surv}}}&\left({\left\{{X}^{(m)},{{y}}_{j}^{(m)},{c}^{(m)}\right\}}_{m = 1}^{M}\right)=\\ &\mathop{\sum }\limits_{i=1}^{M}\left(-{c}^{(m)}\log \left(\;{f}_{{\rm{surv}}}\left({{y}}_{j}^{(m)}| {{\bf{g}}}^{(m)}\right)\right)\right.\\ &+\left(1-{c}^{(m)}\right)\log \left(\;{f}_{{\rm{surv}}}\left({{y}}_{j}^{(m)}-1| {{\bf{g}}}^{(m)}\right)\right)\\ &\left.+\left(1-{c}^{(m)}\right)\log \left(\;{f}_{{\rm{hazard}}}\left({{y}}_{j}^{(m)}| {{\bf{g}}}^{(m)}\right)\right)\right),\end{array}$$

where ${f}_{{\rm{hazard}}}({{y}}_{j}| {\bf{g}})=\,{\rm{Sigmoid}}({\hat{{y}}}_{j})$ is the discrete hazard function and ${f}_{{\rm{surv}}}({{y}}_{j}| {\bf{g}})=\mathop{\prod }\nolimits_{k = 1}^{j}\left(1-{f}_{{\rm{hazard}}}({{y}}_{k}| {\bf{g}})\right)$ is the discrete survival function. Finally, the patient-level risk was defined as the negative sum of all logits^41^, which enables the identification of distinct risk groups and the stratification of patients.

For immune subtype prediction, we adopted the cross-entropy loss defined as

13 $${L}_{{\rm{ce}}}=-\mathop{\sum }\limits_{m=1}^{M}\mathop{\sum }\limits_{k=1}^{K}{{y}}_{k}^{(m)}\log \left({\hat{{y}}}_{k}^{(m)}\right).$$

#### Multimodal fusion via co-attention mechanism

To fuse the patch features from different modalities, we adopted the co-attention mechanism proposed in ref. ^40^. More specifically, given the H&E feature bag ${H}\in {{\mathbb{R}}}^{N\times d}$ and IMC feature bag ${P}\in {{\mathbb{R}}}^{N\times d}$, we guided the feature aggregation of *H* using *P* by calculating the cross-attention

14 $$\begin{array}{rcl}\hat{{{H}}}&=&\,\text{Softmax}\,\left(\frac{{{{W}}}_{q}{P}{{H}}^{\top }{{{W}}}_{k}^{\top }}{\sqrt{d}}\right){{{W}}}_{v}{H}\\ &=&{{{A}}}_{P\to H}{{{W}}}_{v}{H},\end{array}$$

where ${{{W}}}_{q},{{{W}}}_{k},{{{W}}}_{v}\in {{\mathbb{R}}}^{d\times d}$ are learnable weights and ${{{A}}}_{P\to H}\in {{\mathbb{R}}}^{N\times N}$ is the co-attention matrix. Intuitively, the co-attention measures the pairwise similarity for how much an H&E instance **h**_*i*_ attends to the IMC instance **p**_*i*_ for *i* ∈ *N*. Similarly, we can guide the feature aggregation of *P* using *H* via *A*_*H*→*P*_. Each co-attention guided feature bag was input to an attention-based MIL module, which outputted an aggregated WSI-level representation. We concatenated the WSI-level representations from multiple modalities and projected them back to the original feature dimension *d* via a linear layer, resulting in a multimodal WSI-level representation. Then, a classifier *f*(⋅) used this representation to predict the output label $\hat{{y}}$.

#### Implementation and training details

We adopted the original implementation of attention-based MIL^39^ on GitHub at https://github.com/AMLab-Amsterdam/AttentionDeepMIL. We implemented the co-attention mechanism based on the original implementation of MCAT^40^ (https://github.com/mahmoodlab/MCAT). Each WSI was cropped to 256 × 256 non-overlapping patches at ×20 magnification to create bags, where patches with more than 10% non-tissue area were discarded. We used ResNet18 (ref. ^63^) pretrained on pathology-specific datasets using self-supervised learning^64^ to extract features from H&E patches and ResNet50 pretrained on ImageNet^65^ to extract features from IMC patches. Because ResNet18 requires three-channel input, we concatenated IMC images of three different protein markers along the channel dimension: one tumour marker (MelanA) and two immune markers (CD8 and CD20). The dimension of extracted features was 512 for both H&E and IMC patches. We ran the survival and immune subtype prediction for 5-fold cross-validation. The model hyperparameters were set as follows: Adam optimizer with initial learning rate of 1*e*^−4^ (survival) and 5*e*^−5^ (immune subtype), a ReduceLROnPlateau scheme based on validation loss for scheduling and a mini-batch size of 1. The model was trained for 100 epochs with early stopping based on validation loss (survival) and weighted *F*1-score (immune subtype). The data processing and model training were done on an NVIDIA A100 40GB GPU. The deep learning models were trained using PyTorch (v.1.13.1), and the pipeline was implemented in Python (v.3.8.12).

### Reporting summary

Further information on research design is available in the Nature Portfolio Reporting Summary linked to this article.

## Supplementary information

Supplementary Information Supplementary Information on benchmarking on pixel-aligned datasets, TuPro dataset and extended interpretation of results.

Reporting Summary

## Source data

Source Data Figs. 2, 3b and 6c, Extended Data Fig. 2a, and Extended Data Tables 3 and 4 A single file containing all source data with clearly named tabs.

## Figures and Tables

**Extended Data Table 1:**
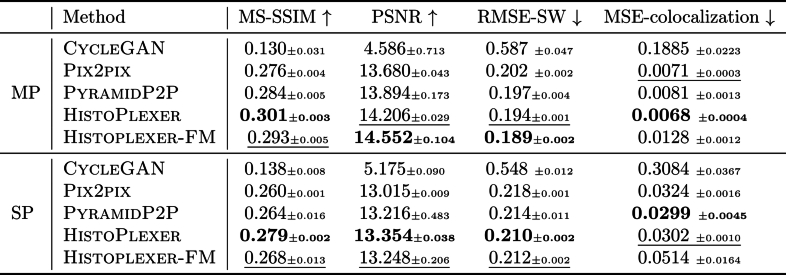
Comparison of HistoPlexer with benchmarks. Comparison of Model Performance against benchmarks using MS-SSIM, PSNR, RMSE-SW and MSE for co-localization patterns for multiplex (MP) and singleplex (SP) settings. *↑* arrow indicates higher values are better. *↓* arrow indicates higher values are better. The best results are highlighted in bold and second best results are underlined

**Extended Data Table 2:**
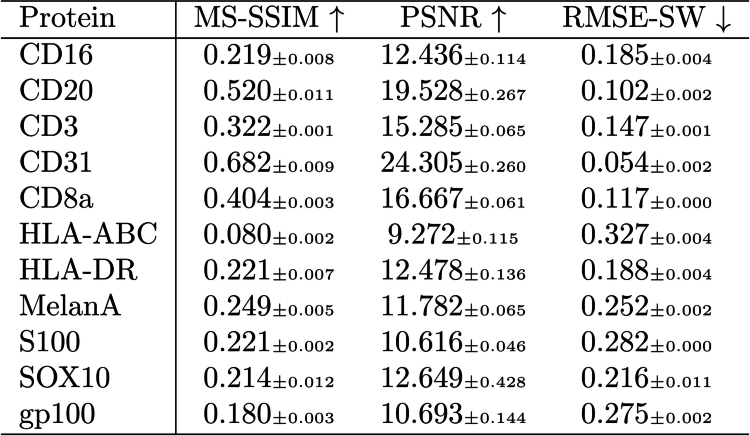
Per marker performance. Performance of HistoPlexer using MSSSIM, PSNR, RMSE-SM metrics for each marker, presented as mean ± standard deviation

**Extended Data Table 3:**
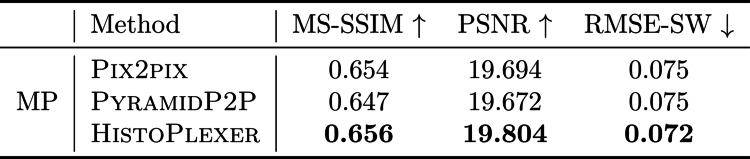
Performance on DeepLIIF-A dataset. Comparison of model performance on the DeepLIIF-A dataset in the multiplex (MP) setting using MS-SSIM, PSNR, and RMSE-SW. Arrows indicate whether higher (*↑*) or lower (*↓*) values are preferable. The best results are highlighted in bold

**Extended Data Table 4:**
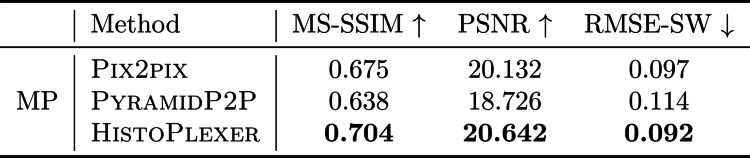
Performance on DeepLIIF-B dataset. Comparison of model performance on the DeepLIIF-B dataset in the multiplex (MP) setting using MS-SSIM, PSNR, and RMSE-SW. Arrows indicate whether higher (*↑*) or lower (*↓*) values are preferable. The best results are highlighted in bold

## Data Availability

The multiplexed WSI images for the Immuno8 and MDSC FixVue panels, generated using Ultivue InSituPlex technology, along with paired H&E images, are publicly available on Hugging Face at https://huggingface.co/datasets/CTPLab-DBE-UniBas/HistoPlexer-Ultivue. The H&E WSIs for TCGA–SKCM were obtained from the GDC Data Portal: https://portal.gdc.cancer.gov/. The Tumor Profiler Study data used for model training contain sensitive clinical information and are subject to ethical and privacy restrictions, preventing unrestricted public release. The data are accessible upon request through the Tumor Profiler Consortium’s portal at https://tumorprofilercenter.ch/contacts. Requests must include a brief scientific proposal outlining the intended use. The Consortium reviews requests typically within four to six weeks and determines the scope, duration and conditions of data access. Approved users are required to comply with data use agreements that restrict data use to specified research purposes and prohibit further sharing without authorization. Source data are provided with this paper.

## Code availability

The source code and trained model weights for HistoPlexer are available at https://github.com/ratschlab/HistoPlexer and on Zenodo at 10.5281/zenodo.15110117 (ref. ^66^).

## References

[1] Hanahan, D. & Weinberg, R. A. Hallmarks of cancer: the next generation. *Cell* **144**, 646–674 (2011). 21376230 10.1016/j.cell.2011.02.013

[2] Hanahan, D. Hallmarks of cancer: new dimensions. *Cancer Discov.* **12**, 31–46 (2022). 35022204 10.1158/2159-8290.CD-21-1059

[3] Egeblad, M., Nakasone, E. S. & Werb, Z. Tumors as organs: complex tissues that interface with the entire organism. *Dev. Cell* **18**, 884–901 (2010). 20627072 10.1016/j.devcel.2010.05.012 PMC2905377

[4] Jackson, H. W. et al. The single-cell pathology landscape of breast cancer. *Nature* **578**, 615–620 (2020). 31959985 10.1038/s41586-019-1876-x

[5] Sobottka, B. et al. Establishing standardized immune phenotyping of metastatic melanoma by digital pathology. *Lab. Investig.* **101**, 1561–1570 (2021). 34446805 10.1038/s41374-021-00653-y PMC8590976

[6] Ptacek, J. et al. Multiplexed ion beam imaging (mibi) for characterization of the tumor microenvironment across tumor types. *Lab. Investig.* **100**, 1111–1123 (2020). 32203152 10.1038/s41374-020-0417-4

[7] Tan, W. C. C. et al. Overview of multiplex immunohistochemistry/immunofluorescence techniques in the era of cancer immunotherapy. *Cancer Commun.* **40**, 135–153 (2020). 10.1002/cac2.12023 PMC7170662 32301585

[8] Windhager, J. et al. An end-to-end workflow for multiplexed image processing and analysis. *Nat. Protoc.* **18**, 3565–3613 (2023). 37816904 10.1038/s41596-023-00881-0

[9] Jin, M.-Z. & Jin, W.-L. The updated landscape of tumor microenvironment and drug repurposing. *Signal Transduct. Target. Ther.* **5**, 166 (2020). 32843638 10.1038/s41392-020-00280-x PMC7447642

[10] Fischer, A. H., Jacobson, K. A., Rose, J. & Zeller, R. Hematoxylin and eosin staining of tissue and cell sections. *Cold Spring Harb. Protoc.* **2008**, 4986 (2008). 10.1101/pdb.prot4986 21356829

[11] Burlingame, E. A. et al. Shift: speedy histological-to-immunofluorescent translation of a tumor signature enabled by deep learning. *Sci. Rep.* **10**, 17507 (2020). 33060677 10.1038/s41598-020-74500-3 PMC7566625

[12] Liu, S. et al. BCI: breast cancer immunohistochemical image generation through pyramid pix2pix. In *Proc. IEEE/CVF Conference on Computer Vision and Pattern Recognition* 1815–1824 (IEEE, 2022).

[13] Liu, S. et al. Unpaired stain transfer using pathology-consistent constrained generative adversarial networks. *IEEE Trans. Med. imaging* **40**, 1977–1989 (2021). 33784619 10.1109/TMI.2021.3069874

[14] Ghahremani, P. et al. An AI-ready multiplex staining dataset for reproducible and accurate characterization of tumor immune microenvironment. In *Proc. International Conference on Medical Image Computing and Computer-Assisted Intervention* (eds Greenspan, H. et al.) 704–713 (Springer, 2023). 10.1007/978-3-031-43987-2_68 PMC10571229 37841230

[15] Pati, P. et al. Accelerating histopathology workflows with generative AI-based virtually multiplexed tumour profiling. *Nat. Mach. Intell.* **6**, 1–17 (2024). 10.1038/s42256-024-00889-5 PMC11415301 39309216

[16] Zhang, R. et al. Mvfstain: multiple virtual functional stain histopathology images generation based on specific domain mapping. *Med. Image Anal.* **80**, 102520 (2022). 35810588 10.1016/j.media.2022.102520

[17] Zhou, Z. et al. Virtual multiplexed immunofluorescence staining from non-antibody-stained fluorescence imaging for gastric cancer prognosis. *eBioMedicine* **107**, 105287 (2024). 39154539 10.1016/j.ebiom.2024.105287 PMC11378090

[18] Irmisch, A. et al. The tumor profiler study: integrated, multi-omic, functional tumor profiling for clinical decision support. *Cancer Cell* **39**, 288–293 (2021). 33482122 10.1016/j.ccell.2021.01.004

[19] Guan, J., Gupta, R. & Filipp, F. V. Cancer systems biology of tcga skcm: efficient detection of genomic drivers in melanoma. *Sci. Rep.* **5**, 7857 (2015). 25600636 10.1038/srep07857 PMC4298731

[20] Ghahremani, P. et al. Deep learning-inferred multiplex immunofluorescence for immunohistochemical image quantification. *Nat. Mach. Intell.* **4**, 401–412 (2022). 36118303 10.1038/s42256-022-00471-x PMC9477216

[21] Li, F., Hu, Z., Chen, W. & Kak, A. Adaptive supervised PatchNCE loss for learning H&E-to-IHC stain translation with inconsistent groundtruth image pairs. In *Proc. Medical Image Computing and Computer Assisted Intervention – MICCAI 2023* (eds Greenspan, H. et al.) 632–641 (Springer, 2023).

[22] Culjak, I., Abram, D., Pribanic, T., Dzapo, H. & Cifrek, M. A brief introduction to OpenCV. In *Proc. 35th International Convention MIPRO* 1725–1730 (IEEE, 2012).

[23] Isola, P., Zhu, J.-Y., Zhou, T. & Efros, A. A. Image-to-image translation with conditional adversarial networks. In *Proc. IEEE Conference on Computer Vision and Pattern Recognition* 1125–1134 (IEEE, 2017).

[24] Wang, Z., Simoncelli, E. P. & Bovik, A. C. Multiscale structural similarity for image quality assessment. In *Proc. 37th Asilomar Conference on Signals, Systems & Computers* (ed. Matthews, M. B.) 1398–1402 (IEEE, 2003).

[25] Detlefsen, N. S. et al. Torchmetrics-measuring reproducibility in pytorch. *J. Open Source Softw.* **7**, 4101 (2022).

[26] Jain, A. K. *Fundamentals of Digital Image Processing* (Prentice-Hall, 1989).

[27] Zhu, J.-Y., Park, T., Isola, P. & Efros, A. A. Unpaired image-to-image translation using cycle-consistent adversarial networks. In *Proc. IEEE International Conference on Computer Vision* 2223–2232 (IEEE, 2017).

[28] Graham, S. et al. Hover-net: simultaneous segmentation and classification of nuclei in multi-tissue histology images. *Med. Image Anal.* **58**, 101563 (2019). 31561183 10.1016/j.media.2019.101563

[29] Zhou, S. et al. Hype: a benchmark for human eye perceptual evaluation of generative models. In *Proc. 33rd Conference on Neural Information Processing Systems* (eds Wallach, H. et al.) 3449–3461 (IEEE, 2019).

[30] Breiman, L. Random forests. *Mach. Learn.* **45**, 5–32 (2001).

[31] Mondello, P. et al. Lack of intrafollicular memory CD4+ T cells is predictive of early clinical failure in newly diagnosed follicular lymphoma. *Blood Cancer J.* **11**, 130 (2021). 34267181 10.1038/s41408-021-00521-4 PMC8282842

[32] Saltz, J. et al. Spatial organization and molecular correlation of tumor-infiltrating lymphocytes using deep learning on pathology images. *Cell Rep.* **23**, 181–193 (2018). 29617659 10.1016/j.celrep.2018.03.086 PMC5943714

[33] Chevrier, S. et al. An immune atlas of clear cell renal cell carcinoma. *Cell* **169**, 736–74918 (2017). 28475899 10.1016/j.cell.2017.04.016 PMC5422211

[34] Herbst, R. S. et al. Predictive correlates of response to the anti-PD-L1 antibody MPDL3280A in cancer patients. *Nature* **515**, 563–567 (2014). 25428504 10.1038/nature14011 PMC4836193

[35] Ji, R.-R. et al. An immune-active tumor microenvironment favors clinical response to ipilimumab. *Cancer Immunol. Immunother.* **61**, 1019–1031 (2012). 22146893 10.1007/s00262-011-1172-6 PMC11028506

[36] Godson, L. et al. Immune subtyping of melanoma whole slide images using multiple instance learning. *Med. Image Anal.* **93**, 103097 (2024). 38325154 10.1016/j.media.2024.103097

[37] Pfannstiel, C. et al. The tumor immune microenvironment drives a prognostic relevance that correlates with bladder cancer subtypes. *Cancer Immunol. Res.* **7**, 923–938 (2019). 30988029 10.1158/2326-6066.CIR-18-0758

[38] Wouters, M. C. & Nelson, B. H. Prognostic significance of tumor-infiltrating B cells and plasma cells in human cancer. *Clin. Cancer Res.* **24**, 6125–6135 (2018). 30049748 10.1158/1078-0432.CCR-18-1481

[39] Ilse, M., Tomczak, J. & Welling, M. Attention-based deep multiple instance learning. In *Proc.**International Conference on Machine Learning* (eds Dy, J. & Krause, A.) 2127–2136 (PMLR, 2018).

[40] Chen, R. J. et al. Multimodal co-attention transformer for survival prediction in gigapixel whole slide images. In *Proc. IEEE/CVF International Conference on Computer Vision* 4015–4025 (IEEE, 2021).

[41] Jaume, G. et al. Modeling dense multimodal interactions between biological pathways and histology for survival prediction. In *Proc. IEEE/CVF Conference on Computer Vision and Pattern Recognition* 11579–11590 (IEEE, 2024).

[42] Antolini, L., Boracchi, P. & Biganzoli, E. A time-dependent discrimination index for survival data. *Stat. Med.* **24**, 3927–3944 (2005). 16320281 10.1002/sim.2427

[43] Chen, R. J. et al. Towards a general-purpose foundation model for computational pathology. *Nat. Med.* **30**, 850–862 (2024). 38504018 10.1038/s41591-024-02857-3 PMC11403354

[44] Mulliqi, N. et al. Foundation models—a panacea for artificial intelligence in pathology? Preprint at https://arxiv.org/abs/2502.21264 (2025).

[45] Bradski, G. The OpenCV Library. *Dr. Dobb’s J.* **120**, 122–125 (2000).

[46] McQuin, C. et al. Cellprofiler 3.0: next-generation image processing for biology. *PLoS Biol.* **16**, 2005970 (2018). 10.1371/journal.pbio.2005970 PMC6029841 29969450

[47] Crowell, H. L. et al. An R-based reproducible and user-friendly preprocessing pipeline for cytof data. *F1000Res.* **9**, 1263 (2020). 36072920 10.12688/f1000research.26073.1 PMC9411975

[48] Otsu, N. A threshold selection method from gray-level histograms. *IEEE Trans. Syst. Man Cybern.* **9**, 62–66 (1979).

[49] Gillies, S. et al. Shapely (2.0.6). *Zenodo*10.5281/zenodo.13345370 (2024).

[50] Macenko, M. et al. A method for normalizing histology slides for quantitative analysis. In *Proc. IEEE International Symposium on Biomedical Imaging: From Nano to Macro* 1107–1110 (IEEE, 2009).

[51] Nan, A., Tennant, M., Rubin, U. & Ray, N. Drmime: differentiable mutual information and matrix exponential for multi-resolution image registration. In *Proc. 3rd Conference on**Medical Imaging with Deep Learning* (eds Arbel, T. et al.) 527–543 (PMLR, 2020).

[52] Ronneberger, O., Fischer, P. & Brox, T. U-net: convolutional networks for biomedical image segmentation. In *Proc. International Conference on Medical Image Computing and Computer-Assisted Intervention* (eds Navab, N. et al.) 234–241 (Springer, 2015).

[53] Mao, X. et al. Least squares generative adversarial networks. In *Proc. IEEE International Conference on Computer Vision* 2794–2802 (IEEE, 2017).

[54] Karnewar, A. & Wang, O. Msg-gan: multi-scale gradients for generative adversarial networks. In *Proc. IEEE/CVF Conference on Computer Vision and Pattern Recognition* 7799–7808 (IEEE, 2020).

[55] Chen, T., Kornblith, S., Norouzi, M. & Hinton, G. A simple framework for contrastive learning of visual representations. In *Proc.**International Conference on Machine Learning* (eds Daumé, H. & Singh, A.) 1597–1607 (PMLR, 2020).

[56] Oord, A. v. d., Li, Y. & Vinyals, O. Representation learning with contrastive predictive coding. Preprint at https://arxiv.org/abs/1807.03748 (2018).

[57] Mescheder, L., Geiger, A. & Nowozin, S. Which training methods for GANS do actually converge? In *Proc. International Conference on Machine Learning* (eds Dy, J. & Krause, A.) 3481–3490 (PMLR, 2018).

[58] Kingma, D. P. & Ba, J. Adam: a method for stochastic optimization. In *Proc. International Conference on Learning Representations* (2015).

[59] Berg, S. et al. Ilastik: interactive machine learning for (bio)image analysis. *Nat. Methods* **16**, 1226–1232 (2019). 31570887 10.1038/s41592-019-0582-9

[60] Pedregosa, F. et al. Scikit-learn: machine learning in Python. *J. Mach. Learn. Res.* **12**, 2825–2830 (2011).

[61] Wagner, J. et al. A single-cell atlas of the tumor and immune ecosystem of human breast cancer. *Cell* **177**, 1330–1345 (2019). 30982598 10.1016/j.cell.2019.03.005 PMC6526772

[62] Zadeh, S. G. & Schmid, M. Bias in cross-entropy-based training of deep survival networks. *IEEE Trans. Pattern Anal. Mach. Intell.* **43**, 3126–3137 (2020). 10.1109/TPAMI.2020.2979450 32149626

[63] He, K., Zhang, X., Ren, S. & Sun, J. Deep residual learning for image recognition. In *Proc. IEEE Conference on Computer Vision and Pattern Recognition* 770–778 (IEEE, 2016).

[64] Ciga, O., Xu, T. & Martel, A. L. Self supervised contrastive learning for digital histopathology. *Mach. Learn. Appl.* **7**, 100198 (2022).

[65] Deng, J. et al. Imagenet: a large-scale hierarchical image database. In *Proc.**IEEE Conference on Computer Vision and Pattern Recognition* 248–255 (IEEE, 2009).

[66] Andani, S. et al. Source data for ‘Histopathology-based protein multiplex generation using deep learning’. *Zenodo*10.5281/zenodo.15110117 (2025). 10.1038/s42256-025-01074-y PMC12364712 40842484

