# Supplementary material for: Histopathology-based protein multiplex generation using deep learning
Source: Nat Mach Intell. 2025 Aug 4;7(8):1292–307. doi: 10.1038/s42256-025-01074-y (PMC12364712; doi:10.1038/s42256-025-01074-y)
Supplement: Supplementary file 2 — Reporting Summary [file 42256_2025_1074_MOESM2_ESM.pdf]

nature portfolio

Corresponding author(s): Rätsch and Koelzer

Last updated by author(s): 27/5/25

Reporting Summary

Nature Portfolio wishes to improve the reproducibility of the work that we publish. This form provides structure for consistency and transparency in reporting. For further information on Nature Portfolio policies, see our Editorial Policies and the Editorial Policy Checklist.

Statistics

For all statistical analyses, confirm that the following items are present in the figure legend, table legend, main text, or Methods section.

n/a	Confirmed	
☒	☐	The exact sample size (*n*) for each experimental group/condition, given as a discrete number and unit of measurement
☒	☐	A statement on whether measurements were taken from distinct samples or whether the same sample was measured repeatedly
☒	☐	The statistical test(s) used AND whether they are one- or two-sided<br>*Only common tests should be described solely by name; describe more complex techniques in the Methods section.*
☒	☐	A description of all covariates tested
☒	☐	A description of any assumptions or corrections, such as tests of normality and adjustment for multiple comparisons
☐	☒	A full description of the statistical parameters including central tendency (e.g. means) or other basic estimates (e.g. regression coefficient) AND variation (e.g. standard deviation) or associated estimates of uncertainty (e.g. confidence intervals)
☐	☒	For null hypothesis testing, the test statistic (e.g. *F*, *t*, *r*) with confidence intervals, effect sizes, degrees of freedom and *P* value noted<br>*Give P values as exact values whenever suitable.*
☒	☐	For Bayesian analysis, information on the choice of priors and Markov chain Monte Carlo settings
☒	☐	For hierarchical and complex designs, identification of the appropriate level for tests and full reporting of outcomes
☐	☒	Estimates of effect sizes (e.g. Cohen's *d*, Pearson's *r*), indicating how they were calculated

Our web collection on statistics for biologists contains articles on many of the points above.

Software and code

Policy information about availability of computer code

Data collection	For qualitative evaluation of HistoPlexer on whole slide image level, we employed Ultivue InSituPlex technology to obtain multiplexed images using the Immuno8 and MDSC FixVue panels for 10 samples from the Tumor Profiler Study. The images were acquired at a resolution of 0.325 µm/pixel.
Data analysis	The data processing and model training was done on NVIDIA A100 40GB GPU. The deep learning models were trained using PyTorch (1.13.1) and the pipeline was implemented in Python (3.8.12).<br>The source code and trained models weights for HistoPlexer are available at https://github.com/ratschlab/HistoPlexer and via Zenodo at https://doi.org/10.5281/zenodo.15110117.

For manuscripts utilizing custom algorithms or software that are central to the research but not yet described in published literature, software must be made available to editors and reviewers. We strongly encourage code deposition in a community repository (e.g. GitHub). See the Nature Portfolio guidelines for submitting code & software for further information.

Data

Policy information about availability of data

All manuscripts must include a data availability statement. This statement should provide the following information, where applicable:

- Accession codes, unique identifiers, or web links for publicly available datasets
- A description of any restrictions on data availability
- For clinical datasets or third party data, please ensure that the statement adheres to our policy

The multiplexed WSI images for the Immuno8 and MDSC FixVue panels, generated using Ultivue InSituPlex technology, along with paired H&E images, are publicly available via Hugging Face at https://huggingface.co/datasets/CTPLab-DBE-UniBas/HistoPlexer-Ultivue. The H&E WSIs for TCGA-SKCM were obtained from the GDC Data Portal: https://portal.gdc.cancer.gov/. The Tumor Profiler study data used for model training contain sensitive clinical information and is subject to ethical and privacy restrictions, preventing unrestricted public release. The data is accessible upon request through the Tumor Profiler Consortium's portal at https://tumorprofilercenter.ch/contacts. Requests must include a brief scientific proposal outlining the intended use. The Consortium reviews requests typically within 4–6 weeks and determines the scope, duration, and conditions of data access. Approved users are required to comply with data use agreements that restrict data use to specified research purposes and prohibit further sharing without authorization.

Research involving human participants, their data, or biological material

Policy information about studies with human participants or human data. See also policy information about sex, gender (identity/presentation), and sexual orientation and race, ethnicity and racism.

Reporting on sex and gender	There was no specific pre-selection done based on sex and gender.
Reporting on race, ethnicity, or other socially relevant groupings	There was no specific pre-selection done based on race, ethnicity or other socially relevant groupings.
Population characteristics	Not relevant for this study.
Recruitment	No patient recruitment was done in the context of this study.
Ethics oversight	The ethics committee of the "Swiss Association of Research Ethics Committees" gave ethical approval for the data from the Tumor Profiler Study used in this work. The Tumor Profiler Study is an approved, observational clinical study (BASEC: 2018-02050, 2018-02052, 2019-01326, 2024-01428).

Note that full information on the approval of the study protocol must also be provided in the manuscript.

Field-specific reporting

Please select the one below that is the best fit for your research. If you are not sure, read the appropriate sections before making your selection.

☒ Life sciences ☐ Behavioural & social sciences ☐ Ecological, evolutionary & environmental sciences

For a reference copy of the document with all sections, see nature.com/documents/nr-reporting-summary-flat.pdf

Life sciences study design

All studies must disclose on these points even when the disclosure is negative.

Sample size	The details on the exact sample size are included in methods and results sections. We used metastatic melanoma samples from the Tumor Profiler Study due to availability of multimodal data, including H&E and IMC images.
Data exclusions	For Tumor Profiler metastatic melanoma patients, the samples where alignment of H&E and IMC images was not possible due to large shifts were excluded.<br>For Ultivue dataset, regions with false signals, particularly those corresponding to hemorrhage, bleeding, or erythrocytes in H&E images, were manually annotated and excluded from analysis.<br>For downstream tasks on TCGA-SKCM dataset, H&E images with low resolution (< 0.3µm/pixel) were excluded from analysis.
Replication	For training of models, the experiments were performed with three random initialization of weights. For downstream tasks, 5-fold of cross validation was performed. Mean/std are reported for the analyses in the manuscript results section.
Randomization	For model training, the dataset was randomly split into training and testing. The split was done at the patient level, stratified by immune phenotype. Details are provided in Methods section.
Blinding	For the Human Eye Perceptual Evaluation, the domain experts were blinded by label of the image (real or generated). Blinding was not done for any other experiment or analysis done in the paper.

Reporting for specific materials, systems and methods

We require information from authors about some types of materials, experimental systems and methods used in many studies. Here, indicate whether each material, system or method listed is relevant to your study. If you are not sure if a list item applies to your research, read the appropriate section before selecting a response.

Materials & experimental systems

n/a	Involved in the study
☐	☒ Antibodies
☒	☐ Eukaryotic cell lines
☒	☐ Palaeontology and archaeology
☒	☐ Animals and other organisms
☒	☐ Clinical data
☒	☐ Dual use research of concern
☒	☐ Plants

Methods

n/a	Involved in the study
☒	☐ ChIP-seq
☒	☐ Flow cytometry
☒	☐ MRI-based neuroimaging

Antibodies

Antibodies used

MelanA
S100
gp100
SOX10
CD3
CD8a
CD20
CD16
CD31
HLA-ABC
HLA-DR

Validation

All antibodies were validated by immunofluorescence imaging prior to isotope-polymer conjugation. Antibodies were tested for cell type and inter-cell location specificity within positive control tissues.

Plants

Seed stocks

Report on the source of all seed stocks or other plant material used. If applicable, state the seed stock centre and catalogue number. If plant specimens were collected from the field, describe the collection location, date and sampling procedures.

Novel plant genotypes

Describe the methods by which all novel plant genotypes were produced. This includes those generated by transgenic approaches, gene editing, chemical/radiation-based mutagenesis and hybridization. For transgenic lines, describe the transformation method, the number of independent lines analyzed and the generation upon which experiments were performed. For gene-edited lines, describe the editor used, the endogenous sequence targeted for editing, the targeting guide RNA sequence (if applicable) and how the editor was applied.

Authentication

Describe any authentication procedures for each seed stock used or novel genotype generated. Describe any experiments used to assess the effect of a mutation and, where applicable, how potential secondary effects (e.g. second site T-DNA insertions, mosiacism, off-target gene editing) were examined.
